# Safety and efficacy of artesunate-amodiaquine combined with either methylene blue or primaquine in children with falciparum malaria in Burkina Faso: A randomized controlled trial

**DOI:** 10.1371/journal.pone.0222993

**Published:** 2019-10-10

**Authors:** Margarida Mendes Jorge, Lucienne Ouermi, Peter Meissner, Guillaume Compaoré, Boubacar Coulibaly, Eric Nebie, Johannes Krisam, Christina Klose, Meinhard Kieser, Albrecht Jahn, Guangyu Lu, Umberto D`Alessandro, Ali Sié, Frank Peter Mockenhaupt, Olaf Müller

**Affiliations:** 1 Heidelberg Institute of Global Health, University Hospital, Heidelberg, Baden-Württemberg, Germany; 2 Centre de Recherche en Santé de Nouna, Nouna, Kossi, Burkina Faso; 3 Department of Paediatrics, University Hospital, Ulm, Germany; 4 Institut of Medical Biometry and Informatics, University Hospital, Heidelberg, Baden-Württemberg, Germany; 5 Medical College, Yangzhou University, Yangzhou, Jiangsu, China; 6 MRC Unit The Gambia at the London School of Hygiene and Tropical Medicine, London, London, United Kingdom; 7 Institute of Tropical Medicine and International Health, Charité-Universitätsmedizin Berlin, Berlin, Germany; University of Tübingen, GERMANY

## Abstract

Artemisinin resistance is threatening global efforts for malaria control and elimination. Primaquine (PQ) and methylene blue (MB) are gametocytocidal drugs that can be combined with artemisinin-based combination therapy (ACT) to reduce malaria transmission, including resistant strains. Children (6–59 months) with uncomplicated falciparum malaria in Burkina Faso were treated with artesunate-amodiaquine (AS-AQ) and randomized to MB (15 mg/kg/day for 3 days) or PQ (0.25 mg/kg at day 2) with the aim to show non-inferiority of the MB regimen with regard to haematological recovery at day 7 (primary endpoint). MB-AS-AQ could not be shown to be non-inferior to PQ-AS-AQ (mean Hb difference between treatment groups on day 7 was -0.352, 95% CI -0.832–0.128, p = 0.0767), however, haemoglobin recovery following treatment was alike in the two study arms (day 7: mean 0.2±1.4 g/dl vs. 0.5±0.9 g/dl, p = 0.446). Occurrence of adverse events was similar in both groups, except for vomiting, which was more frequent in the MB than in the PQ arm (20/50 vs 7/50, p = 0.003). Adequate clinical and parasitological response was above 95% in both groups, but significantly more asexual parasites were cleared in the MB arm compared to the PQ arm already on day 1 (48/50, 96%, vs 40/50, 80%, p = 0.014). Moreover, *P*. *falciparum* gametocyte prevalence and density were lower in the MB arm than in the PQ arm, which reached statistical significance on day 2 (prevalence: 2/50, 4%, vs 15/49, 31%, p<0.001; density: 9.6 vs 41.1/μl, p = 0.024). However, it should be considered that PQ was given only on day 2. MB-ACT appears to be an interesting alternative to PQ-ACT for the treatment of falciparum malaria. While there is a need to further improve MB formulations, MB-ACT may already be considered useful to reduce falciparum malaria transmission intensity, to increase treatment efficacy, and to reduce the risk for resistance development and spread.

**Trial registration:** ClinicalTrials.gov NCT02851108.

## Introduction

Malaria remains the most important parasitic disease worldwide [1]. Globally, malaria morbidity and mortality have been much reduced since 2000, but progress has stalled in recent years [2]. The greatest burden of malaria remains in sub-Saharan African (sSA) [1, 3] where most malaria deaths occur in young children with limited access to health services [1, 3, 4].

Artemisinin-based combination therapy (ACT) is now the standard first-line treatment for uncomplicated falciparum malaria in all endemic areas [2, 5]. However, resistance against artemisinin compounds and its partner drugs has emerged in South-East Asia [5], and there is concern that it may spread or develop *de novo* in other endemic areas, including sSA [2, 5]. A potential strategy to prevent and delay the emergence and spread of artemisinin resistance is to add another anti-malarial drug (triple therapy) to standard ACT [6, 7]. Combining a gametocytocidal drug such as primaquine (PQ) or methylene blue (MB) with an ACT has furthermore been shown to reduce transmission of *P*. *falciparum* parasites, including drug-resistant parasites [8–11].

PQ is known to be effective against *P*. *falciparum* gametocytes but is not widely used in endemic areas because of concerns on the risk of haemolytic anaemia in individuals with glucose-6-phosphate dehydrogenase (G6PD) deficiency [12]. Nevertheless, a single, low dose of PQ (0.25 mg base/kg) is considered safe and is now recommended by the World Health Organization (WHO), even without G6PD testing [2, 12]. In randomised controlled trials (RCTs) carried out in sSA, single low dose PQ regimens were associated with significantly reduced but clinically irrelevant reductions in haemoglobin levels as compared to regimens lacking PQ [13, 14].

MB is the oldest synthetic antimalarial drug [15]. In historical studies, MB was consistently shown to be safe and highly effective in all endemic areas and against all human malaria species. However, the use of MB against malaria was terminated after alternative synthetic antimalarials without colouring properties entered the market [16]. More recent studies have confirmed the pluripotent activity of MB and its efficacy against *P*. *falciparum* and *P*. *vivax* asexual parasites, demonstrated synergy in combination with artemisinin derivatives, and confirmed a substantial gametocytocidal effect in *P*. *falciparum* infection [8, 9, 16]. Most of these studies were carried out in West Africa and combined different MB doses with different ACTs, including artesunate-amodiaquine (AS-AQ) and dihydroartemisinin-piperaquine [8, 10].

## Materials and methods

### Study design and participants

The study was designed as a mono-centre, two-arm randomised controlled phase II non-inferiority study and was conducted in the research zone of the Centre de Recherche en Santé de Nouna (CRSN) in north-western Burkina Faso [17]. Malaria is highly endemic and transmission seasonal (July-December), with most cases occurring during or shortly after the rainy season [18].

The primary objective was to determine the safety of MB as compared to PQ when combined with AS-AQ for the treatment of uncomplicated falciparum malaria in children 6–59 month old. Secondary objectives were to determine the treatment efficacy and to assess its acceptance by the parents/care givers.

The study was not blinded given that MB results in blue urines. Nevertheless, all lab technicians were blinded to the origin of samples to be analysed.

Children attending Nouna Hospital with confirmed uncomplicated falciparum malaria were recruited if they fulfilled all the following inclusion criteria: 6–59 months old; parasite density: 2.000–100.000 parasites/μl; axillary temperature ≥ 37.5°C or a history of fever during the last 24 hours; weight ≥ 6 kg; and written informed consent of parents or care givers. Children were excluded if they had at least one of the following criteria: Severe malaria [19]; mixed malaria infections; haemoglobin < 7 g/dl; any disease other than malaria; history of a previous, significant adverse reaction or known allergy to one or more of the study drugs; antimalarial treatment within the last seven days; and treatment with serotonin reuptake inhibitors or with drugs known to inhibit the liver enzymes cytochrome 2A6 and/or cytochrome 2C8.

### Study procedures

#### Patient recruitment and randomization

Fever measurement points equipped with rapid diagnostic tests (RDT) for malaria (SD Bioline, Abbott, USA) were established in a rotating manner in different quarters of Nouna town. Eligible children were transferred to Nouna Hospital for further examination and final inclusion into the study. After informed consent, children were randomised by the study physician to one of the two study arms according to a pre-established randomization list using block randomization, with each treatment allocation concealed in opaque sealed envelopes that were opened only after the patient’s recruitment.

#### Treatment

AS-AQ (IPCA, India) was administered according to three weight groups (6.0–8.9 kg: 25 mg AS + 67.5mg AQ; 9.0–17.9 kg: 50 mg AS + 135 mg AQ; >17.9 kg: 100 mg AS + 270 mg AQ). 2 mg MB mini-tablets (Pharbil Waltrop GmbH, Germany) were administered once daily (together with AS-AQ) at a daily dose of 15 mg/kg for 3 days (days 0–2), and according to four weight groups (6.0–8.9 kg: 100 mg; 9.0–12.9 kg: 150 mg; 13.0–16.9 kg: 200 mg; >16.9 kg: 250 mg). Depending on the age of the study children, tablets were dissolved or crushed and administered with water. MB was given on a spoon with local food (e. g. banana, honey). PQ tablets (Sanofi, India) were administered at the last day of AS-AQ treatment (day 2). Tablets were dissolved in water and given with a plastic cup or spoon, followed by a cup of orange juice, according to four weight groups (6.0–8.9 kg: 2 mg PQ; 9.0–12.9 kg: 3 mg PQ; 13.0–16.9 kg: 4 mg PQ; >16.9 kg: 5 mg PQ). All treatments were directly observed. Full treatment was re-administered once if vomiting occurred within half an hour of treatment.

Children with an axillary temperature ≥ 38.5°C received standard doses of paracetamol (10 mg/kg every 6 hours) until fever subsided. Study children developing severe malaria during the trial were admitted to the Nouna Hospital and treated with artesunate intravenously according to national guidelines.

#### Patients’ follow up

After treatment (days 0–2), the parent/care giver was asked to return with the child for scheduled visits on days 3, 7, 14 and 28, or if any symptoms occurred. For each visit, a physical examination was performed by the study clinicians, vital signs were recorded, and axillary temperature measured with an electronic thermometer. Adverse events (AEs) and serious adverse events (SAEs) were recorded and monitored throughout the study. Rescue treatment for recurrent malaria infections identified during the 28-day follow up as well as for severe malaria was according to local national guidelines.

#### Laboratory procedures

Dried blood spots on filter paper, for G6PD and parasite genotyping, were collected on days 0, 7, 14 and 28, and whenever a child presented to the study team with symptoms during follow-up. The laboratory work was conducted in the laboratory of the *CRSN* which is integrated in the Nouna Hospital. Quality control of all malaria slides was done at the malaria laboratory of the MRC Unit The Gambia. All molecular biology diagnostics were undertaken at the Institute of Tropical Medicine and International Health, Charité, Berlin. DNA was extracted using commercial kits (QIAamp, Qiagen, Germany). Recrudescence was differentiated from re-infection by comparing PCR-generated *P*. *falciparum msp1* and *msp2* amplicons at day 0 (before treatment) and at the time of recurrent infection. G6PD typing (codons 202 & 376; rs1050828 & rs1050829) was performed by melting curve analysis applying commercial primers and probes (TIB Molbiol, Berlin, Germany).

#### Outcome

The primary outcome was haematological recovery (haemoglobin changes during follow-up); more specifically haemoglobin difference between day 7 and day 0 was the primary endpoint. Secondary outcomes were *P*. *falciparum* gametocyte prevalence and density by microscopy, the area under the curve (AUC) of *P*. *falciparum* gametocyte density, the incidence of observed and self-reported AEs and SAEs during follow-up, fever and parasite clearance rates as well as the rates of adequate clinical and parasitological response (ACPR), early treatment failure (ETF), late treatment failure (LTF) and late parasitological failure (LPF) [20]. The reported acceptance of the different treatment regimens by parents or care givers on day 14 was also assessed. Rates of ACPR, LTF and LPF were assessed with and without polymerase chain reaction (PCR)-correction for recrudescence/reinfection.

The AUC was calculated using the linear trapezoidal rule taking the measurements at day 0, 1, 2, 3, 7, 14 and 28 into account.

### Statistical analysis

#### Analysis sets

The primary efficacy analysis was based on the full analysis set (FAS) according to the intention-to-treat (ITT) principle [21]. Additionally, an evaluation of the primary outcome was performed in the per protocol (PP) set to emphasize the importance of the PP analysis in non-inferiority trials [22]. The PP set included all patients of the ITT set without major protocol deviations (defined as either a violation of inclusion or exclusion criteria, informed consent obtained after randomisation, or vomiting after the repeated drug intake at either day 0, day 1, or day 2).

#### Sample size calculation

The sample size calculation was based on the primary comparison of the mean haemoglobin change between day 0 and day 7. Assuming a standard deviation of 1.1722 in both groups [9], 50 patients per arm would be needed to demonstrate non-inferiority between study arms with a clinically relevant non-inferiority margin of δ = -0.7, at 80% power, a one-sided significance level of 2.5%, and allowing 10% loss to follow-up. The sample size was calculated using ADDPLAN v6.1.

#### Primary endpoint

The hypotheses for the primary efficacy analysis were

$$H_{0}:\;\mu_{MB}-\mu_{PQ}\leq \delta \;vs.\;H_{1}:\;\mu_{MB}-\mu_{PQ}>\delta$$

(δ = -0.7, non-inferiority margin), where μ_MB_ and μ_PQ_ are the mean differences in haemoglobin between day 7 and baseline for the MB and PQ group, respectively. Non-inferiority was tested at a significance level of α = 0.025 using a one-sided t-test and the mean difference between groups was determined with a 95% confidence interval. Missing data for the primary outcome variable was replaced using multiple imputations [23]. A complete case analysis was done as sensitivity analysis.

#### Secondary endpoints

Secondary endpoints were assessed by means of descriptive statistics. Since the p-values derived are solely of an exploratory nature, no adjustment for multiple testing was done.

Haemoglobin differences compared to baseline were analysed using a linear mixed model for repeated measures adjusting for baseline haemoglobin, treatment group, and time. In addition, separate linear models were fitted for each time point adjusting for baseline haemoglobin. The gametocyte densities and AUC were compared between groups using Mann-Whitney-U tests, and gametocyte prevalence using Cochrane-Mantel-Haenszel tests adjusted for baseline gametocyte prevalence. ACPR, ETF, LCF, LPF, fever and parasite clearance rates were compared using chi-squared tests. The acceptance of the different treatment regimens was compared using the Mantel-Haenszel chi-squared test for ordinal outcomes.

AEs and SAEs were tabulated; absolute and relative frequencies with 95% confidence intervals were calculated. Possible differences between the treatment groups were tested using the chi-squared test.

## Results

### Baseline data

**Fig 1:**
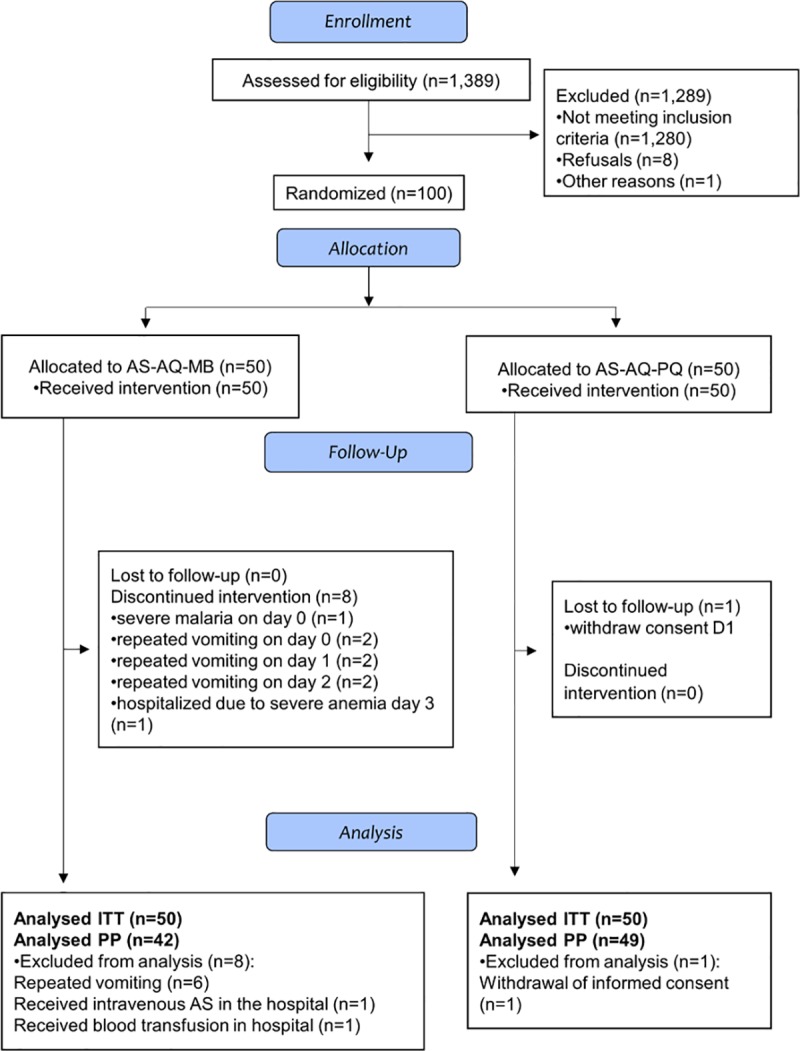
Consort flow chart.

Between the 3^rd^ of October 2016, and the 7^th^ of November, 2016, 100 children were recruited in the study out of 1,389 febrile children screened (1,289 excluded: 1,113 RDT negative, 2 parasite densities >100.000/μl and 161 < 2.000/μl, 2 severe malnutrition, 2 >59 months old, 8 refusals, and 1 from outside the study area). Those enrolled were randomly assigned to receive, besides AS-AQ, either MB (n = 50) or PQ (n = 50) (Fig 1).

Overall, baseline characteristics between the two study arms were similar (Table 1). The mean age was 40 months, and half of the children were females. The mean duration of the current malaria episode was two days, and about a third of children had already received paracetamol. The mean haemoglobin concentration was 9.92 g/dl, and the mean asexual parasitaemia was 26.846 parasites/μl. Most patients (87/100, 87%) did not have *P*. *falciparum* gametocytes at baseline. Full G6PD deficiency (hemizygous or homozygous for Gd^A-^) was observed in 14/100 (14%) patients and heterozygosity in 16/100 (16%).

**Table 1:** Baseline characteristics between study arms.

Variable	AS-AQ-MB N = 50	AS-AQ-PQ N = 50	Total N = 100
Age [months]			
Mean +/- SD	42.34 +/-12.34	38.20 +/-13.93	40.27 +/-13.26
Median	43	39	42
Min, Max	13, 59	10, 59	10, 59
Female gender			
female	24/50 (48%)	25%0 (50%)	49/100 (49%)
Weight [kg]			
Mean +/- SD	12.91 +/-3.16	12.18 +/-2.98	12.55 +/-3.08
Median	13	12	12
Min, Max	7.5, 22	7, 18	7, 22
Length of current disease episode [days]			
Mean +/- SD	1.94 +/-1.32	1.96 +/-1.28	1.95 +/-1.29
Median	1	1.5	1
Min, Max	1, 7	1, 6	1, 7
Prior treatment of current disease episode			
none	34 (68%)	37 (74%)	71 (71%)
Paracetamol	16 (32%)	12 (24%)	28 (28%)
Traditional medicine	0 (0%)	1 (2%)	1 (1%)
Any other prior illnesses within last 7 days	4/50 (8%)	2/50 (4%)	6/100 (6%)
Temperature [°C]			
Mean +/- SD	37.8 +/-0.81	37.79 +/-0.79	37.8 +/-0.79
Median	37.60	37.6	37.6
Min, Max	36, 40.5	36.7, 40.8	36, 40.8
Haemoglobin [g/dl]			
Mean +/- SD	10.16 +/-1.62	9.68 +/-1.42	9.92 +/-1.53
Median	10.5	9.65	10.05
Min, Max	7, 13.6	7, 12.6	7, 13.6
*P*. *falciparum* asexual parasite density			
Mean +/- SD	30373.6 +/-32808.1	23318.8 +/-25199.72	26846.2 +/-29319.34
Median	16040	11820	13460
Min, Max	2080, 98600	2040, 96400	2040, 98600
*P*. *falciparum* gametocytes	5/50 (10%)	8/50 (16%)	13/100 (13%)
*P*. *falciparum* gametocytes parasite density (for those patients with gametocytes)			
Mean +/- SD	200 +/-123.29	170 +/-110.58	181.54 +/-111.49
Median	200	120	120
Min, Max	80, 400	40, 360	40, 400
G6PD genotype			
deficient (hemi- or homozygous for Gd^A-^)	7/50 (14.0%)	7/50 (14.0%)	14/100 (14.0%)
heterozygous	11/50 (22.0%)	5/50 (10.0%)	16/100 (16.0%)
non-deficient	32/50 (64.0%)	38/50 (76.0%)	70/100 (70.0%)

#### Follow-up data

All 100 study children were included in the ITT analysis. For the PP analysis, 42 in the MB arm and 49 in the PQ arm were included (Fig 1). In the PQ arm, one child was excluded from the PP set due to withdrawal of consent at day 1. In the MB arm, 2 children were excluded from the PP set, one developed severe malaria at day 0 and was treated with intravenous AS, and one developed severe anaemia at day 3, and was treated with blood transfusion in hospital. Vomiting the study medication within ½ hours after treatment was significantly more frequent in the MB (20/50, 40%) than in the PQ (7/50, 14%) arm (p = 0.003). Six children (all in the MB arm) discontinued treatment after repeated vomiting on day 0 (n = 2), day 1 (n = 2), and day 2 (n = 2) and were treated with artemether-lumefantrine (Fig 1).

All parents/care givers of children treated with MB confirmed the appearance of blue urines from the beginning of the treatment until day 3; none of the parents of children treated with PQ reported blue urines.

### Primary analysis and haemoglobin development

**Fig 2:**
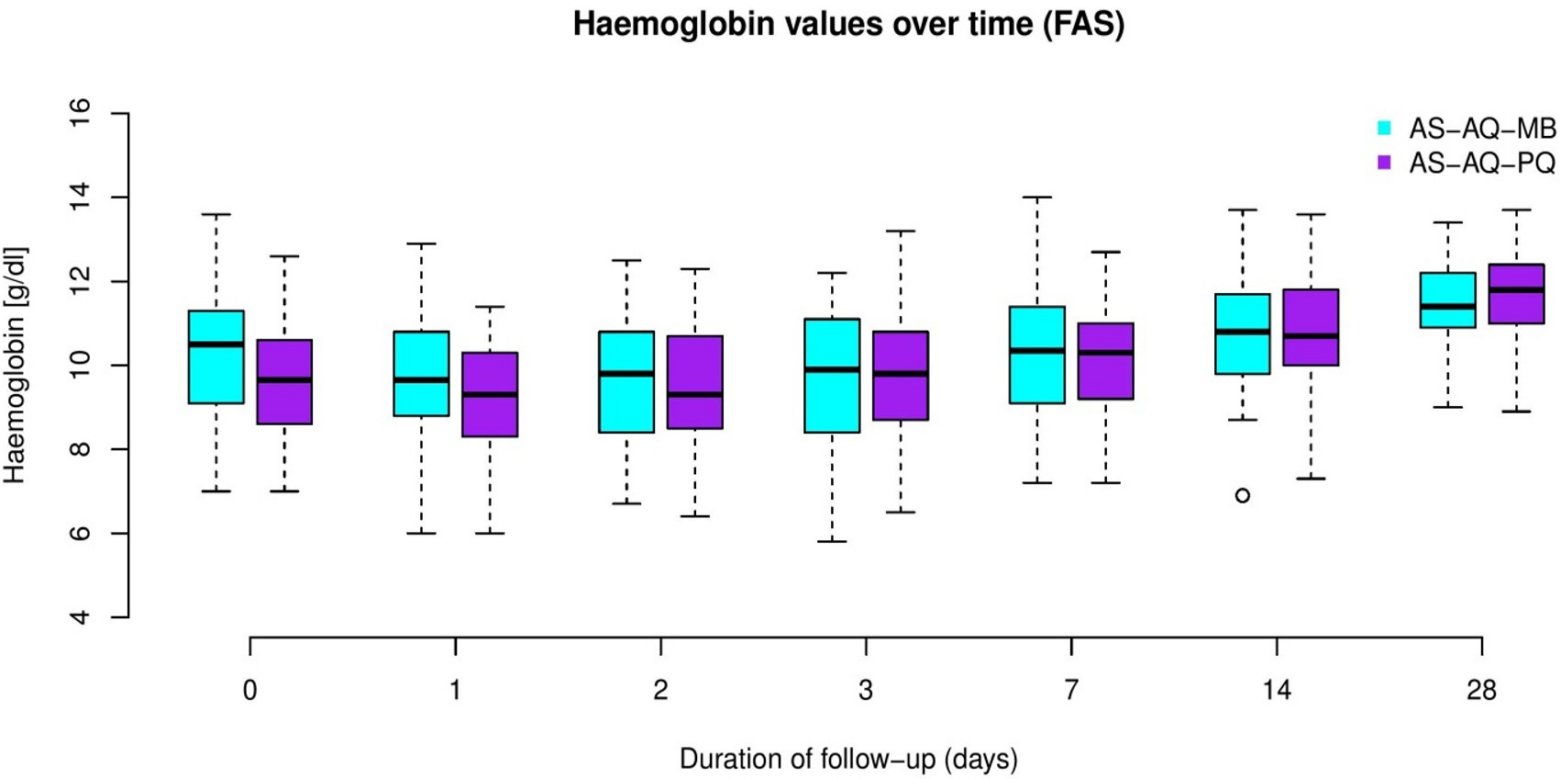
Boxplots of haemoglobin values by study group over time (ITT analysis).

Fig 2 shows the haemoglobin values by study group over the whole follow-up (ITT analysis). Children in the PQ arm started with slightly lower haemoglobin values than those in the MB arm.

The mean Hb difference between treatment groups on day 7 was -0.352 (95% confidence interval: -0.832 and 0.128) (p = 0.0767) for one-sided non-inferiority test, indicating that it was not possible to demonstrate non-inferiority according to the chosen non-inferiority margin of δ = -0.7. The complete case analysis of the ITT set and the PP set yielded comparable results for the mean difference (ITT: -0.363, CI = [-0.843;0.117], p = 0.0832, PP: -0.353, CI = [-0.847;0.141], p = 0.831). These results indicate a slightly worse haematological recovery for MB-AS-AQ compared to PQ-AS-AQ, which could neither be shown to be non-inferior nor inferior to PQ, since all confidence intervals contain both the non-inferiority margin of -0.7 and the value 0.

Table 2 shows the differences between baseline and follow-up haemoglobin values (ITT analysis) adjusted for baseline values. Children in the MB arm showed consistently higher differences in Hb values between day 0 and days of follow up than children in the PQ arm; this was close to statistical significance on day 3 (mean -0.4±1.11 g/dl vs. 0.12±1.10 g/dl, p = 0.062). On day 7 (primary endpoint), the haemoglobin difference between groups was slightly lower (mean 0.18±1.42 g/dl vs. 0.54±0.94 g/dl, p = 0.446).

**Table 2:** Comparison of haemoglobin differences between baseline and follow-up days by study group and controlled for baseline values (ITT analysis).

Variable	AS-AQ-MB N = 50	AS-AQ-PQ N = 50	Total N = 100	p-value*
Haemoglobin difference (Day 1—Day 0)				0.462
N	50	49	99	
Mean +/- SD	-0.64 +/-0.88	-0.41 +/-1.03	-0.52 +/-0.96	
Median	-0.60	-0.30	-0.50	
Min, Max	-3.00, 0.99	-3.50, 1.20	-3.50, 1.20	
Haemoglobin difference (Day 2—Day 0)				0.230
N	50	49	99	
Mean +/- SD	-0.52 +/-1.03	-0.21 +/-0.87	-0.37 +/-0.96	
Median	-0.45	-0.30	-0.40	
Min, Max	-3.30, 1.20	-2.70, 1.30	-3.30, 1.30	
Haemoglobin difference (Day 3—Day 0)				0.062
N	50	49	99	
Mean +/- SD	-0.40 +/-1.11	0.12 +/-1.10	-0.14 +/-1.13	
Median	-0.30	0.20	0.00	
Min, Max	-2.80, 1.40	-2.60, 2.10	-2.80, 2.10	
Haemoglobin difference (Day 7—Day 0)				0.446
N	50	49	99	
Mean +/- SD	0.18 +/-1.42	0.54 +/-0.94	0.36 +/-1.21	
Median	0.20	0.50	0.40	
p25, p75	-0.60, 1.30	-0.10, 1.20	-0.30, 1.30	
Min, Max	-3.00, 2.90	-1.40, 3.10	-3.00, 3.10	
95% CI Mean	[-0.23;0.58]	[0.27;0.81]		
Haemoglobin difference (Day 14—Day 0)				0.295
N	50	49	99	
Mean +/- SD	0.61 +/-1.68	1.14 +/-1.13	0.87 +/-1.45	
Median	0.80	1.00	0.80	
Min, Max	-5.00, 5.20	-0.50, 3.80	-5.00, 5.20	
Haemoglobin difference (Day 28—Day 0)				0.125
N	50	49	99	
Mean +/- SD	1.37 +/-1.51	1.99 +/-1.16	1.68 +/-1.38	
Median	1.35	2.00	1.70	
Min, Max	-2.10, 4.40	0.00, 4.50	-2.10, 4.50	

*p-values based on linear models adjusting for baseline haemoglobin.

The PP analysis provided similar results.

#### Adverse events

One child in the MB arm developed repeated vomiting of the study medication on day 0 and was considered as a case of severe malaria as it was a rather young child (15 months) with high parasitaemia (14.600/μl), and as it was very weak (unable to take medicine orally or to drink anything). A 22 months old child (G6PD normal) in the MB arm developed severe anaemia as defined in the present study (haemoglobin value dropped from 7.1 g/dl on day 0 to 5.8 g/dl on day 3), was hospitalized and received a blood transfusion, afterwards the child fully recovered. This was considered as a SAE; there were no further SAEs and no deaths in the study.

Table 3 shows the non-serious AEs by group (ITT analysis). The most frequent AE were respiratory and gastro-intestinal symptoms, and there were no significant differences between the two study groups.

**Table 3:** Number and characteristics of non-severe adverse events (ITT analysis).

	AS-AQ-MB (N = 50)			AS-AQ-PQ (N = 50)			Total (N = 100)			
	F	N	P	F	N	P	F	N	P	p-value*
Total number of non-serious adverse events	58	33		67	28		125	61		
Respiratory symptoms	25	20	61%	35	20	71%	60	40	66%	1.000
Gastro-intestinal symptoms	19	14	42%	16	13	46%	35	27	44%	0.822
Pain	0			3	3	11%	3	3	5%	0.079
Skin symptoms	6	6	18%	2	2	7%	8	8	13%	0.140
Urinary tract symptoms	1	1	3%	1	1	4%	2	2	3%	1.000
Fever	2	2	6%	4	3	11%	6	5	8%	0.646
Anaemia symptoms	0			0			0			------
Lymphadenopathy	0			1	1	4%	1	1	2%	0.315
General weakness/tiredness	5	4	12%	4	4	14%	9	8	13%	1.000
Others	0			0			0			------

F = number of events, N = number of subjects with at least one event, P = number of subjects with the respective event divided by the total number of subjects experiencing adverse events

*p-values based on chi-squared test

#### Clearance of fever and asexual parasites

By day 3, 97% of all patients were afebrile (ITT analysis). It should be noted that 35 patients had only a history of fever at inclusion and not a raised body temperature; they were excluded from the fever clearance analysis.

Table 4 shows the asexual parasite clearance data. In the ITT analysis, significantly more patients had cleared their parasites by day 1 in the MB than in the PQ arm (48/50, 96.0% vs 40/50, 80.0%; p = 0.014). By day 2, all patients were parasite-free and remained so at day 3. The difference in asexual parasite clearance between the two study groups was even more pronounced in the PP analysis.

**Table 4:** Parasite clearance during follow-up (ITT analysis).

Variable	AS-AQ-MB N = 50	AS-AQ-PQ N = 50	Total N = 100	p-value*
Parasite clearance until day 1	48/50 (96.0%)	40/50 (80.0%)	88/100 (88.0%)	0.014
Parasite clearance until day 2	50/50 (100.0%)	49/49 (100.0%)	99/99 (100.0%)	------
Parasite clearance until day 3	50/50 (100.0%)	49/49 (100.0%)	99/99 (100.0%)	------

*p-value based on chi-squared test

#### Treatment efficacy

There were no differences in the ITT analysis between the two study groups in terms of PCR-uncorrected (MB: 89.4%, 42/47; PQ: 91.8%, 45/49) (p = 0.677) and PCR-corrected (MB: 95.7%, 45/47; PQ: 95.9%, 47/49) (p = 0.966) ACPR rate (Table 5). Analysis of the PP population gave similar results.

**Table 5:** Efficacy outcome by study group.

Variable	AS-AQ-MB N = 50	AS-AQ-PQ N = 50	Total N = 100	p-value*
Early treatment failure (ETF)				
no	47 (100.0%)	49 (100.0%)	96 (100.0%)	
missing	3	1	4	
Late clinical failure (LCF)				
no	47 (100.0%)	48 (98.0%)	95 (99.0%)	0.325
yes	0 (0.0%)	1 (2.0%)	1 (1.0%)	
missing	3	1	4	
Late clinical failure (LCF) adjusted for reinfection				
no	47 (100.0%)	48 (98.0%)	95 (99.0%)	0.325
yes	0 (0.0%)	1 (2.0%)	1 (1.0%)	
missing	3	1	4	
Late parasitological failure (LPF)				
no	42 (89.4%)	46 (93.9%)	88 (91.7%)	0.424
yes	5 (10.6%)	3 (6.1%)	8 (8.3%)	
missing	3	1	4	
Late parasitological failure (LPF) adjusted for reinfection				
no	45 (95.7%)	48 (98.0%)	93 (96.9%)	0.533
yes	2 (4.3%)	1 (2.0%)	3 (3.1%)	
missing	3	1	4	
Adequate clinical and parasitological response (ACPR)				
no	5 (10.6%)	4 (8.2%)	9 (9.4%)	0.677
yes	42 (89.4%)	45 (91.8%)	87 (90.6%)	
missing	3	1	4	
Adequate clinical and parasitological response (ACPR) adjusted for reinfection				
no	2 (4.3%)	2 (4.1%)	4 (4.2%)	0.966
yes	45 (95.7%)	47 (95.9%)	92 (95.8%)	
missing	3	1	4	

*p-value based on chi-squared test

#### Effects on gametocytes

**Fig 3:**
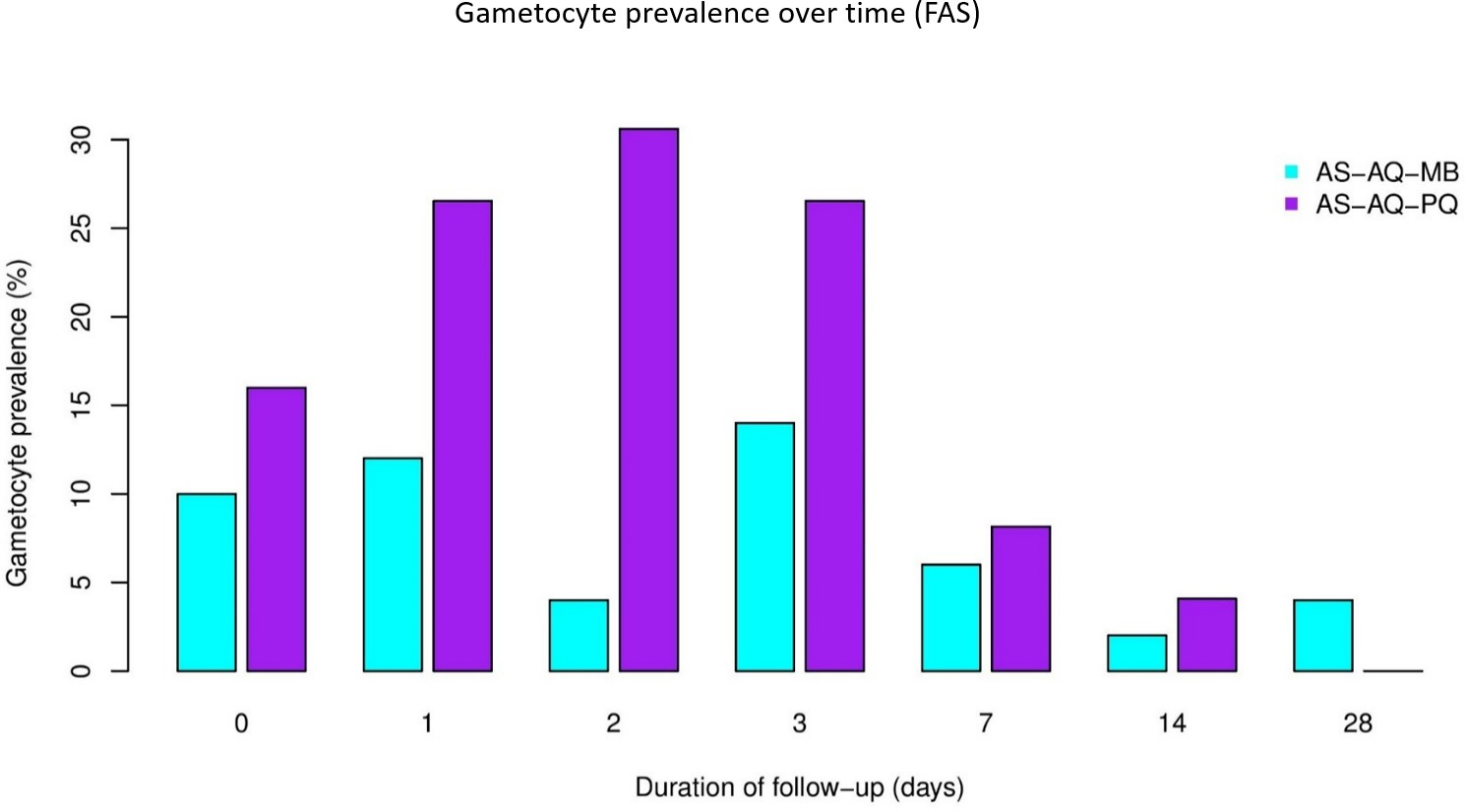
Gametocyte prevalence during follow up by group.

Fig 3 shows the gametocyte prevalence during follow-up by study group (FAS). Gametocyte prevalence was lower in the MB than in the PQ arm during the whole follow-up, except at day 28.

Table 6 shows the effect of the different treatments on gametocyte prevalence, density and AUC. At baseline, the prevalence of *P*. *falciparum* gametocytes in the MB and PQ arms was 10% and 18%, respectively. During follow-up and adjusting for baseline values, the gametocyte prevalence was consistently lower until day 14 in the MB than in the PQ arm, although it reached statistical significance only at day 2. At baseline, the mean density of *P*. *falciparum* gametocytes in the MB and PQ arm was 20.0±70.1/μl and 27.2±75.6/μl, respectively. During follow-up and adjusting for baseline values, gametocyte density was consistently lower until day 14 in the MB than in the PQ arm; the difference was statistically significant at days 1 and 2. The mean AUC of *P*. *falciparum* gametocyte density was lower in the MB than in the PQ arm, but the difference was not statistically significant (p = 0.165). However, it has to be considered that the differences between the two arms do not involve a direct comparison between MB and PQ on respective follow-up days as different application schemes were used in the two study arms.

**Table 6:** Gametocyte prevalence, density and AUC by study group.

Variable	AS-AQ-MB N = 50	AS-AQ-PQ N = 50	Total N = 100	p-value*
Gametocytes at day 0	5/50 (10.0%)	8/50 (16.0%)	13/100 (13.0%)	0.372
Gametocytes at day 1	6/50 (12.0%)	13/49 (26.5%)	19/99 (19.2%)	0.108
Gametocytes at day 2	2/50 (4.0%)	15/49 (30.6%)	17/99 (17.2%)	<0.001
Gametocytes at day 3	7/50 (14.0%)	13/49 (26.5%)	20/99 (20.2%)	0.189
Gametocytes at day 7	3/50 (6.0%)	4/49 (8.2%)	7/99 (7.1%)	0.762
Gametocytes at day 14	1/50 (2.0%)	2/49 (4.1%)	3/99 (3.0%)	0.614
Gametocytes at day 28	2/50 (4.0%)	0/49 (0.0%)	2/99 (2.0%)	0.175
Gametocyte density per μl at day 0				0.399
N	50	50	100	
Mean +/- SD	20.00 +/-70.10	27.20 +/-75.57	23.60 +/-72.61	
Min, Max	0.00, 400.00	0.00, 360.00	0.00, 400.00	
Gametocyte density per μl at day 1				0.042
N	50	49	99	
Mean +/- SD	8.64 +/-33.20	50.61 +/-121.75	29.41 +/-90.86	
Min, Max	0.00, 200.00	0.00, 600.00	0.00, 600.00	
Gametocyte density per μl at day 2				0.024
N	50	49	99	
Mean +/- SD	9.60 +/-57.46	41.14 +/-86.72	25.21 +/-74.74	
Min, Max	0.00, 400.00	0.00, 400.00	0.00, 400.00	
Gametocyte density per μl at day 3				0.354
N	50	49	99	
Mean +/- SD	13.92 +/-45.97	48.98 +/-145.53	31.27 +/-108.36	
Min, Max	0.00, 272.00	0.00, 920.00	0.00, 920.00	
Gametocyte density per μl at day 7				0.459
N	50	49	99	
Mean +/- SD	3.68 +/-15.27	6.69 +/-24.95	5.17 +/-20.58	
Min, Max	0.00, 80.00	0.00, 120.00	0.00, 120.00	
Gametocyte density per μl at day 14				0.436
N	50	49	99	
Mean +/- SD	1.28 +/-9.05	5.06 +/-24.80	3.15 +/-18.60	
Min, Max	0.00, 64.00	0.00, 128.00	0.00, 128.00	
Gametocyte density per μl at day 28				0.197
N	50	49	99	
Mean +/- SD	1.60 +/-8.08	0.00 +/-0.00	0.81 +/-5.77	
Min, Max	0.00, 48.00	0.00, 0.00	0.00, 48.00	
Area under the curve of P. falciparum gametocyte density versus time (including day 1 and 2)				0.165
N	50	49	99	
Mean +/- SD	107.92 +/-196.17	318.04 +/-778.61	211.92 +/-572.12	
Median	0.00	16.00	0.00	
Min, Max	0.00, 800.00	0.00, 4656.00	0.00, 4656.00	

*p-value based on Mann-Whitney-U-test for density values (based on the difference from baseline) and the AUC, chi-squared test for prevalence values at baseline, and CMH tests adjusted for baseline prevalence for prevalence rates at follow-up

#### Acceptance

Overall, at day 14 more than 80% of parents or care givers reported a good acceptance of the study medication. However, acceptance was lower in the AS-AQ-MB compared to the AS-AQ-PQ schedule (Table 7).

**Table 7:** Acceptance of the different treatment regimens by parents or care givers.

Variable	AS-AQ-MB N = 50	AS-AQ-PQ N = 50	Total N = 100	p-value*
How acceptable was the study medication				0.052
good	39 (83%)	46 (93.9%)	85 (88.5%)	
acceptable	5 (10.6%)	3 (6.1%)	8 (8.3%)	
bad	3 (6.4%)	0 (0%)	3 (3.1%)	
missing	3	1	4	

Good: the child has accepted the treatment without problem and I highly recommend the study medication; Acceptable: the child took the treatment after some insistence, but I recommend the study drug; Bad: the child took the medicine after a threat and I would recommend the study medication only after it has been reformulated

*p-value based on Cochrane-Mantel-Haenszel test

## Discussion

The main findings from this study are that treatment with MB-ACT compared to PQ-ACT is associated with [1] a higher rate of vomiting of the study medication, [2] a slightly higher difference in haemoglobin values but no difference in the rate of AEs during follow-up, [3] a more rapid clearance of asexual *P*. *falciparum* parasites, and [4] a lower prevalence and density of *P*. *falciparum* gametocytes during follow-up.

That MB is having a strong bitter taste is well known since it has been used as an antimalarial some 130 years ago [16]. Providing MB with food and sweets (e.g. honey) has been shown to improve acceptance and to reduce vomiting in young children, but not sufficiently [15, 24, 25]. Taste-masking through coated tablets has also been tried in older children and–together with the provision of the treatment with food–has led to an ameliorated but not sufficiently better acceptance [26]. Malaria treatment with MB formulated as mini-tablets is considered as safe in very young children and has led to further improvement of the acceptability in children but was still associated with higher rates of vomiting compared to control groups in previous trials as well as in this trial [27, 28]. It remains to be shown if the development of taste-masked mini tablets will further increase the acceptability of MB in children. However, treatment acceptability of MB is generally much better in older children and in adults [25, 28, 29].

MB belongs to the large group of drugs–many of them anti-malarials–which are a risk factor for haemolysis in patients with G6PD deficiency. With regard to such risk in sSA, a synopsis of four RCTs comparing haemolysis risk of MB-based combination therapy (n = 844) with control therapy (n = 161) showed a small but significant effect of MB on the reduction of haemoglobin values in the treatment of children with malaria in West Africa, which was however considered not to be of clinical relevance [30]. PQ is also deemed unsafe for patients with G6PD deficiency, but single low-dose PQ is largely considered as safe and is recommended by WHO as a standard method for the reduction of *P*. *falciparum* transmission intensity [2, 12]. In this study, MB given over three days was not shown to be non-inferior compared to single low dose PQ, but the small difference in haemoglobin trajectory may be attributed to MB been given over three days and in a relatively high dose. Moreover, the reduction of haemoglobin values associated with this dose of MB has been considered to be clinically non-significant in a pooled analysis of existing studies [30].

MB has been shown *in vitro* to act synergistic with artemisinin derivates, which has already been supported by data from previous clinical studies in sSA [8, 26, 31]. The results from this study confirm this important finding and thus support the value of MB combined with artemisinins in the treatment of falciparum malaria. A more rapid parasite clearance may lead to an overall better clinical outcome including fewer patients progressing to severe malaria.

MB has recently been shown in a preclinical study to be the most effective antimalarial for reducing *P*. *falciparum* gametocytes, and this effect has been confirmed by a clinical study in Burkina Faso [32, 33]. Moreover, it could be shown that this translates into a significant reduction of *P*. *falciparum* transmission [27]. The findings from this study provide further support for a superiority of the chosen MB-ACT regimen compared to standard PQ-ACT regarding reduction of *P*. *falciparum* gametocytes. If this effect would lead to a reduction of transmission intensity under malaria elimination program conditions needs to be studied in large community-based trials.

Artemisinin resistance has rapidly been spreading in recent years in South-East Asia [2, 5]. If artemisinin resistance occurs in sSA, a public health disaster comparable to what has happened some 20 years ago when chloroquine resistance entered the continent would be unavoidable [34]. MB is a pluripotent drug which has independent modes of action against *P*. *falciparum* parasites and appears to have a very low potential for resistance development itself [16]. Using triple therapy for malaria treatment by adding MB to existing ACT regimens has been proposed to be a potential solution to the threat of artemisinin resistance development and spread [7, 35]. Thus, the further development of MB-ACT for large-scale use in endemic areas should be considered a promising approach for the achievement of further progress towards global malaria control and elimination.

This study has some limitations. First, MB was provided as uncoated mini tablets. If these mini tablets would have been given in a taste-masked formulation, the occurrence of early vomiting might have been much reduced. Second, as MB was given over three days from day 0 until day 2 and PQ was given as single dose treatment on day 2, the effects of the two regimens (including the different drug volumes applied) are not fully comparable and this may partly explain the observed differences in vomiting rates and acceptance as well as the tendency to a difference in haemoglobin development during follow-up. Moreover, at least the early effects of the different treatments on gametocyte prevalence and density are clearly influenced by MB and PQ being given in different schedules. Finally, non-inferiority could not be demonstrated for the primary comparison of the study. In the sample size calculation, we assumed no difference between the two treatment arms regarding the mean haemoglobin change between day 0 and day 7, while we observed a difference of -0.352 between MB and PQ in our trial. This indicates that we based our sample size calculations on a too optimistic treatment effect estimate, thus leading to a reduced power for the primary analysis of our trial.

## Conclusions

Although non-inferiority could not be demonstrated, adding MB to ACT has been shown to have a number of potential benefits regarding secondary outcomes of the trial. While there is a need to further modify the MB formulation to improve acceptability, adding MB to existing ACT should be considered as potentially useful to reduce *P*. *falciparum* transmission intensity, to increase treatment efficacy, and to reduce the risk for development and spread of malaria parasites resistant against ACT.

## Supporting information

S1 Checklist Consort checklist. (PDF)

S1 Protocol Clinical trial protocol. (PDF)

S1 Dataset Anonymized data set. (ZIP)

## Abbreviations

ACPRm: Adequate clinical and parasitological response
ACT: Artemisinin-based combination therapy
AE: Adverse Event
AS-AQ: Artesunate-amodiaquine
AUC: Area under the curve
CRSN: Centre de Recherche en Santé de Nouna
ETF: Early treatment failure
FAS: Full analysis set
G6PD: Glucose-6-phosphate dehydrogenase
GCP: Good Clinical Practice
ITT: Intention-to-treat
LPF: Late parasitological failure
LTF: Late treatment failure
MB: Methylene blue
PCR: Polymerase chain reaction
PQ: Primaquine
sSA: Sub-Saharan African
RCT: Randomised controlled trial
RDT: Rapid diagnostic tests
SAE: Serious adverse event
WHO: World Health Organisation
