# Supplementary material for: Safety and efficacy of artesunate-amodiaquine combined with either methylene blue or primaquine in children with falciparum malaria in Burkina Faso: A randomized controlled trial
Source: PLoS One. 2019 Oct 10;14(10):e0222993. doi: 10.1371/journal.pone.0222993 (PMC6786573; doi:10.1371/journal.pone.0222993)
Supplement: S1 Protocol — (PDF) [file pone.0222993.s002.pdf]

Version 1

Safety of artesunate-amodiaquine combined with methylene blue or primaquine for falciparum malaria treatment in African children: A randomised controlled trial

Principal investigator: Prof. Dr. med. Olaf Müller, MPH
Institute of Public Health
Ruprecht-Karls-University Heidelberg
INF 324, 69120 Heidelberg, Germany
Tel.: +49 6221 56 5035
E-mail: olaf.mueller@urz.uni-heidelberg.de

Co-PIs: Dr. Boubacar Coulibaly
Nouna Health Research Centre
P.O. Box 34, Nouna, Burkina Faso
Tel.: +226 537055
E-mail: boubacoulibaly@hotmail.com

Dr. Ali Sie, MD
Nouna Health Research Centre
P.O. Box 34, Nouna, Burkina Faso
Tel.: +226 537055
E-mail: alisie.crsn@fasonet.bf, sieali@yahoo.fr

PD Dr. Peter Meissner, MD, MSc Trop Paed
Department of Pediatrics, Ulm University
Eythstraße 24, 89075 Ulm, Germany
Tel.: +49 731 50057083
E-mail: peter.meissner@uniklinik-ulm.de

Biometrician: Prof. Dr. Meinhard Kieser
Institute of Medical Biometry and Informatics
INF 130.3, 69120 Heidelberg, Germany

Tel: +49 6221 56 4140
E-mail: meinhard.kieser@imbi.uni-heidelberg.de

Members of study team:

Eric Nebie, MD
Nouna Health Research Centre
P.O. Box 34, Nouna, Burkina Faso
Tel.: +226 537055
E-mail: neric83@yahoo.fr

Margarida Jorge, Pharmacist
Institute of Public Health
Ruprecht-Karls-University Heidelberg
INF 324, 69120 Heidelberg, Germany
Tel.: +49 6221 56 4885
E-mail: margaridamjorge@gmail.com

Christina Klose, Data Manager
Institute of Medical Biometry and Informatics
INF 130.3, 69120 Heidelberg, Germany
Tel: +49 6221 56 4140
E-mail: klose@imbi.uni-heidelberg.de

Johannes Krisam, Statistician
Institute of Medical Biometry and Informatics
INF 130.3, 69120 Heidelberg, Germany
Tel: +49 6221 56 4140
E-mail: krisam@imbi.uni-heidelberg.de

Prof. Dr. Frank Mockenhaupt
Institute of Tropical Medicine and International Health
Charité-Universitätsmedizin Berlin, Campus Virchow-Klinikum
Augustenburger Platz 1, 13353 Berlin, Germany
Tel: +49 (0)30 450 565721

E-mail: frank.mockenhaupt@charite.de

Prof. Dr. med. Heiner Schirmer
Biochemie-Zentrum Heidelberg
Ruprecht-Karls-University Heidelberg
INF 328, 69120 Heidelberg
Tel.: +49 6221 54 4165/4175
E-mail: heiner.schirmer@gmx.de

Sponsor Medical School, Ruprecht-Karls-University Heidelberg, Germany

1 Protocol summary

Title: Safety of artesunate-amodiaquine combined with methylene blue or with primaquine for falciparum malaria treatment in African children: A randomised controlled trial.

Design: Mono-center, two arms, open randomised controlled non-inferiority study in children with uncomplicated falciparum malaria in Burkina Faso.

Phase: Phase II.

Objectives: The primary objective of this trial is to investigate the safety of artesunate (AS) – amodiaquine (AQ) - methylene blue (MB) compared to AS – AQ - primaquine (PQ) in young children with uncomplicated falciparum malaria in Burkina Faso

Population: Children aged 6-59 months with uncomplicated falciparum malaria from Nouna Hospital in north-western Burkina Faso.

Sample size: 100 patients (50 per study arm).

Treatment: The group AS-AQ-MB will receive once daily a fixed dose AS-AQ formulation combined with once daily MB (15 mg/kg) over a three days period. The control group will

receive once daily a fixed dose AS-AQ over three days combined with a single dose of PQ on day 2 (0.25 mg/kg).

Endpoints: Primary endpoint is the haemoglobin value on day 7 compared to baseline. Secondary endpoints are adverse events (AE), adequate clinical and parasitological response (ACPR) rate (PCR-corrected for recrudescence), as well as gametocyte prevalence and density.

Study duration and dates: The study will be implemented during the rainy season of the year 2016 from June until November.

2 Abbreviations

MB	Methylene blue
AQ	Amodiaquine
AS	Artesunate
PQ	Primaquine
ACT	Artemisinin-based combination therapy
SAE	Serious adverse event
AE	Adverse event
SSA	Sub-Saharan African
CRF	Case Report Form
CRSN	Nouna Health Research Centre
ACPR	Adequate clinical and parasitological response
ETF	Early treatment failure
LCF	Late clinical failure
LPF	Late parasitological failure

Definitions

Term	Definition
Baseline values	Directly after the written informed consent a blood sample will be taken for the baseline measurements (laboratory baseline values).
Age	The age is calculated using the date of informed consent and date of birth. If the day of the birth date is unknown, it is set to 15. If day and month are missing the 1st of July is assumed to be the date of birth.
D0, D1, D2, D3, D7, D14, D28	For the definition of study days a modification of the latest WHO definition is used (WHO 2003).<br>D0= calendar day of the first treatment, D1= first calendar day after the calendar day of first treatment. These definitions are used for the endpoints ETF, LCF, LPF, ACPR, gametocyte prevalence, density and infectivity, and fever or parasite clearance time. D14 and D28 are defined as the 14th and 28th day after first drug intake (tolerance +/- 2 days).
ETF	• Danger signs or severe malaria on D1, D2 or D3 + parasitemia

Term	Definition
	• Parasitemia on D2 > D0 count irrespective of axillary temperature<br>• Parasitemia on D3 > 25% D0 count irrespective of axillary temperature
LCF	• Development of danger signs or severe malaria after D3 in the presence of parasitemia, without meeting any of the criteria of ETF<br>• Presence of parasitemia + axillary temperature ≥37.5°C on any day from D4 to D28, without previously meeting any criteria of ETF
LPF	• Presence of parasitemia on any day from D7 to D28 + axillary temperature <37.5°C, without previously meeting any of the criteria of ETF
ACPR	• No parasites on D28 irrespective of axillary temperature without previously meeting any of the criteria of ETF, LCF or LPF
Recrudescence	Re-emerging of the same *P. falciparum* strain from D7 to D28 according to PCR genotyping
Reinfection	New infection from D3 to D28 according to PCR genotyping
AEs	The adverse event (=AE) defined in chapter 9 is used. Blue urine is expected for all children in the MB treatment group. Only AEs after initiation of study treatment will be tabulated.
SAE	The definition of serious adverse event (SAE) is given in chapter 9
Haemolysis	Any drop in haemoglobin (Hb) of >2.5 g/dl within 24 hours
Severe acute haemolysis	Hb ≤ 5 g/dl or received blood transfusion according to clinical judgement of study physician.
Severe acute neurological disorder	Acute change in consciousness<br>≥ 2 convulsions

3 Introduction and study rationale

Malaria

Malaria remains the globally most important parasitic disease (Greenwood et al. 2005). The greatest burden of malaria is in sub-Saharan African (SSA) where it has been estimated that 40% of fever episodes are caused by malaria and where children regularly experience several malaria episodes each year (Greenwood et al. 2005, Müller et al. 2001). Most malaria deaths occur in young children of rural SSA areas with little access to health services (Snow et al. 1999, Greenwood et al. 2005, Müller et al. 2003).

Treating malaria with a combination of effective drugs has become a new paradigm in malaria control, with the particular aim to delay and possibly reverse the development of drug resistance through different mechanisms (Nosten and Brasseur 2002). Artemisinin-based combination therapy (ACT) has proved highly effective and has now become the standard first-line treatment for falciparum malaria in all endemic areas (International Artemisinin Study Group 2002, WHO 2015). As resistance against artemisinin compounds has recently started to emerge in South-East Asia, there is a clear need to develop alternative malaria drug combinations (Noedl et al. 2008). Adding another anti-malarial with a short half-life such as methylene blue to standard ACT could be a strategy to prevent artemisinin resistance development (Müller et al. 2009). Moreover, adding a gametocytocidal drug to ACT reduces the probability of transmission of *P. falciparum* parasites including drug-resistant parasites. Such effects are particularly important for malaria treatment in countries or regions aiming at malaria elimination (WHO 2015).

Methylene blue

Methylene blue (MB) is a water-soluble dye used for a long time in industry and medicine. In humans, the drug is rapidly and widely distributed throughout the body (Schirmer et al. 2003; Wainwright & Amaral 2005; Schirmer et al. 2011). The drug is well absorbed from the gastrointestinal tract and partly monodemethylated to azure B in hepatocytes. Maximal plasma concentrations are reached 2 hours following oral administration, and the plasma half-life is about 20 hours (Walter-Sack et al. 2009). Renal excretion of MB and azure B (in oxidised blue or reduced uncoloured forms) is the main elimination pathway (DiSanto and Wagner 1972, Peter et al. 2000). Known side effects of MB are a bitter taste, a mild and self-limiting dysuria, and a blue colouration of the urine (Schirmer et al. 2011, Müller et al. 2013). In contrast to its obvious toxicity when applied intravenously at doses >10 mg/kg, the oral application of MB is usually well tolerated. Even oral doses up to 17 g (5x 100-200 mg per day) were well tolerated

in early malaria studies (Guttmann and Ehrlich 1891). In a historical case series of 40 children with malaria from Brazil, oral doses of 20-50 mg/kg per day given over days to weeks have also been well tolerated (Ferreira 1893).

MB has been used against malaria for more than 100 years (Guttmann und Ehrlich 1891, Ehrlich 1913). Its further use against human malaria is not well documented, thus only observational data supported its efficacy, particularly in malaria cases found refractory to quinine (Ehrlich 1913, Dale 1956). MB is a registered drug in most countries for a number of indications such as treatment of methaemoglobinaemia (Schirmer et al. 2011). It is also used for the prevention of ifosfamid-induced encephalopathy in human cancer treatment. For these indications, MB is usually given intravenously 2-6 times per day at a dose of 1-2 mg/kg (Mansuri and Lurie 1993, Zulian et al. 1995, Aeschlimann et al. 1998, Pelgrims et al. 2000). The use of MB in malaria treatment may also be beneficial in preventing methaemoglobinaemia and thus tissue hypoxia (Anstey et al. 1996). Another potentially clinically relevant effect is an increase in blood pressure in patients with septic shock due to a blockade of NO-dependent guanylate cyclase (Galili et al. 1997).

The interest in MB as an antimalarial drug was reactivated when *P. falciparum* glutathione reductase was identified as a new drug target (Färber et al. 1998, Sarma et al. 2003, Schirmer et al. 2003). MB inhibits the glutathione reductase of the malaria parasite and less the human enzyme; like its major catabolite, azure B, MB is a subversive redox-cycling substrate, and similar to 4-aminoquinolines it seems to also interact with the polymerization of haem to hemozoin (Kanzok et al. 2000, Davioud-Charvet et al. 2001). In the food vacuole reduced MB inhibits metHb degradation by reducing MetHb to Hb. Indeed there seem to be even more but not yet fully understood mechanisms how the pluripotent MB interacts with the parasite which makes resistance development rather unlikely (Wainwright & Amaral 2005). MB has been shown to be very effective in inhibiting *P. falciparum* in culture and this has been confirmed in the mouse model (Vennerstrom et al. 1995).

In a series of clinical studies recently conducted in Burkina Faso, oral MB (4-24 mg/kg/day given for three days) was shown to be safe and effective in the treatment of uncomplicated falciparum malaria when combined with other antimalarials (Mandi et al. 2005, Meissner et al. 2005, Meissner et al. 2006), but acted rather slowly against the asexual parasites (Bountogo et al. 2010). Also a single oral dose of 500 mg MB was well tolerated in healthy German adults (Walter-Sack et al. 2009). Finally, a synopsis of data from more than 1000 children with and without glucose 6-phosphate-dehydrogenase (G6PD) deficiency, which were enrolled into clinical trials with MB in West Africa showed no clinically relevant drop of

haemoglobin during follow-up (Müller et al. 2013). Given the strong *in vitro* and *in vivo* effect on *P. falciparum* gametocyte reduction (Adjalley et al. 2011, Coulibaly et al. 2009), the potential synergy with artemisinin drugs (Akoachere et al. 2005), and the low potential for resistance development, MB has been considered a potentially useful partner drug for artemisinin-based combination therapies (ACT), particularly in regions where elimination is the final goal (Müller et al. 2009). This is supported by the findings from the first MB-ACT trial conducted in 2011 in Burkina Faso (Coulibaly et al. 2015).

Primaquine

8-aminoquinolons are chemically related to methylene blue (MB) and seem to function through a NADPH oxidation process (Rosenthal 2000). However, how exactly primaquine (PQ) works is unknown.

PQ is the only registered drug against the hypnozoites of *P. vivax* and is also effective against the asexual forms and the gametocytes of this parasite. However, it is not widely used in endemic areas due to serious adverse events when given at high dose and/or for a prolonged time to patients with G6PD deficiency (Recht et al. 2014). In particular, the potential of PQ to cause life-threatening drop of haemoglobin levels is feared, which may occur when the G6PD activity is below 10% (Recht et al. 2014). Although G6PD prevalence in SSA is high (ranging from 5-30% in the male population), the prominent G6PD deficiency type in SSA (A-) usually has a high residual enzyme activity and is thus not considered very dangerous (Bousema & Drakeley 2011, Recht et al. 2014).

In areas of low malaria transmission, it was formerly recommended that a single dose of primaquine (0.75 mg base/kg; 45 mg adult dose) be added to ACT in acute falciparum malaria to block malaria transmission. Review of studies of transmission-blocking activity based on the infectivity of patients or volunteers to anopheles mosquitoes, and of haemolytic toxicity in G6PD deficient subjects, suggests that a lower primaquine dose (0.25 mg base/kg) would be safer and equally effective. WHO now recommends that this lower dose can be deployed together with ACTs without G6PD testing wherever use of a specific gametocytocide is indicated (Recht et al. 2014).

4 Trial objectives and purpose

The overall goal of the underlying research project is to develop a MB-based first-line drug combination regimen against uncomplicated falciparum malaria in SSA.

The primary objective of this study is:

- To study the safety of the triple combination AS-AQ-MB compared to AS-AQ-PQ in the treatment of uncomplicated falciparum malaria in young African children

The secondary objective of this study is:

- To study the efficacy of this MB-based triple combination in comparison with standard ACT-PQ in the treatment of uncomplicated falciparum malaria in young African children

Justification: ACT combined with single low-dose PQ is the standard regimen used against falciparum malaria in areas were transmission reduction is the main goal. There is *in vitro* and *in vivo* evidence for MB acting synergistic with artemisinin drugs by both altering the redox system of *P. falciparum*. Moreover, there is also *in vitro* and *in vivo* evidence for MB acting against both the young and the old stages of gametocytes. Increased efficacy of the antimalarial regimen would likely also reduce gametocyte production. Finally, adding MB to standard ACTs may further protect the artemisinin component against resistance development by its independent modes of action together with the lowering of transmission of pre-resistant parasites.

Definition of outcomes

Primary endpoint:

- Change in haemoglobin values on day 7 of follow-up compared to baseline

Secondary endpoints:

- *P. falciparum* gametocyte prevalence and density on days 1, 2, 3, 7, and 28 of follow-up (microscopic determination)
- The area under the curve of *P. falciparum* gametocyte density versus time
- Incidence of observed and self-reported adverse events over the 28 days observation period
- Fever and parasite clearance time
- Haematological recovery, i.e. haemoglobin concentrations during follow-up
- Adequate clinical and parasitological response (ACPR) as assessed by microscopy rate (PCR-corrected for recrudescences) over a 28 days observation period
- Rates of early treatment failures (ETF), late treatment failures (LCF) and late parasitological failures (LPF) (PCR-corrected for recrudescences) over the 28 day observation period
- Acceptance of the different treatment regimens by mothers/caretakers assessed on day 14

5 Design of the trial

The study is designed as a mono-centre, two-arm randomised controlled phase II non-inferiority study in north-western Burkina Faso. As MB leads to blue urine, the study will be open label but with blinding for the laboratory technicians who are responsible for haemoglobin measurements and parasite determination in blood smears and PCR samples (observer blinded study).

Study procedures:

100 children aged 6-59 months with uncomplicated falciparum malaria will be included into the study. All study children will be followed up for 28 days.

After all data have been collected, double entered and cleaned at the study site, the database will be closed. Data analysis will be carried out in Germany through independent statisticians. Report writing and publication will take place in Burkina Faso and Germany involving key researchers from all collaborating partners. Finally, the policymakers in Burkina Faso will be informed through specific workshops and the implications of the study results on the respective malaria control policies will be discussed.

All study procedures will be conducted according to Good Clinical Practice (GCP) guidelines.

The study will take place in the study area of the CRSN in Burkina Faso, where highly experienced staff (physicians, nurses, fieldworker, and laboratory technicians) for the conduct of clinical studies is already available.

Children will become recruited from Nouna town. Fever measurement points will become established in a rotating manner in the different quarters of the town. Children found eligible for the study will be transferred to the study unit in the Nouna Hospital for further examination and final inclusion into the study.

Randomisation and blinding

The randomisation numbers will be allocated to the two groups using the random number generator of the validated software SAS®. Sealed envelopes are produced and labelled with the randomization number. Inside of the sealed envelope, a data sheet defines the group allocation of the patient with the respective randomization number. On enrolment in the study, each patient receives the randomisation envelope in consecutive order of inclusion in the study. Basic characteristics of the patient and day of randomisation are documented on the data sheet in order to allow a check whether the randomization scheme was strictly observed.

Due to the colouration of urine through MB, blinding of patients is not possible. However, all lab technicians examining the blood samples for haemoglobin values, malaria parasites and other study-relevant aspects will be blinded to treatment regimen.

Duration of the trial

The study will be implemented between June and November 2016. Follow-up of study subjects will be 28 days.

6 Detailed study procedures

Study area

The area is located in the Nouna Health District in rural north-western Burkina Faso. The study area is highly endemic for malaria, with most transmission taking place during and shortly after the rainy season, which usually lasts from June until October. The area is populated mainly by subsistence farmers of the Bobo and Marka ethnic groups. Study children will be recruited from the outpatient department of the district hospital in Nouna town. The field work will be coordinated by the *Centre de Recherche en Santé de Nouna* (CRSN), which is situated in Nouna town. The laboratory work will be conducted in the laboratory of the CRSN.

Sample size

The sample size calculation is based on the primary comparison of the mean change in haemoglobin (day 7 – baseline) between the two treatment groups. 50 patients per arm are needed to assess non-inferiority of AS-AQ-MB to AS-AQ-PQ with a power of 80% at a one-sided significance level of 2.5%, with allowance for 10% loss to follow-up and with the use of a clinically relevant non-inferiority margin of δ=0.7, assuming a standard deviation of 1.1722 in both groups (estimate based on own unpublished data from Coulibaly et al. 2015). Thus, the overall number of study patients is 100. Sample size calculation was performed using ADDPLAN v6.1.

Inclusion criteria

- 6-59 months old children (male and female)
- Weight $\geq$ 6 kg
- Uncomplicated malaria caused by *P. falciparum*
- Asexual parasites $\geq$ 2 000/µl and $\leq$ 100 000/µl
- Axillary temperature $\geq$ 37.5°C or a history of fever during the last 24 hours
- Burkinabe nationality
- Permanent residence in the study area with no intention of leaving during the surveillance period
- Written informed consent of parents or care takers

Exclusion criteria

- Severe malaria (WHO 2000)
- Mixed malaria infection

- Vomiting (≥ 3 times within 24 hours before the visit)
- Any apparent significant disease, including severe malnutrition
- A history of a previous, significant adverse reaction or known allergy to one or more of the study drugs
- Anaemia (haemoglobin < 7 g/dl)
- Treated in the same trial before
- All modern antimalarial treatment prior to inclusion (last seven days)
- Therapy with serotonin reuptake inhibitors (e.g. citalopram, escitalopram, fluoxetine, Paroxetine, Sertraline)
- Simultaneous participation in another investigational study
- Treatment with other investigational drugs
- Patients with known HIV/AIDS disease
- Therapy with drugs known to inhibit the liver enzymes cytochrome 2A6 (e.g. methoxsalen, pilocarpine, tranylcypromine) and/or cytochrome 2C8 (e.g. trimethoprim, ketoconazole, ritonavir, saquinavir, lopinavir, gemfibrozil, montelukast)

Withdrawal criteria

Participants may be withdrawn from the study at their guardians´ request. In case of vomiting after the repeated drug intake (one repetition only) of the first drug ingestion, the participant will be withdrawn from the study.

Description of the treatments

All study drugs will be given according to standard operation procedures (SOP) established at the CRSN. The group AS-AQ-MB will receive once daily one tablet of the fixed dose AS-AQ (Coarsucam®, Sanofi Aventis, taken from the quality-controlled stock of the Ministry of Health) using three weight groups (6.0 – 8.9 kg: 25 mg AS + 67.5mg AQ; 9.0-17.9 kg: 50 mg AS + 135 mg AQ; >17.9 kg: 100 mg AS + 270 mg AQ), combined with once daily MB (15 mg/kg) mini-tablets using four weight groups (6.0-8.9 kg: 100 mg MB; 9.0-12.9 kg: 150 mg MB; 13.0-16.9 kg: 200 mg MB; >16.9 kg: 250 mg MB) given over a three days period. Each mini-tablet contains 2 mg MB. Coarsucam® tablets will be taken with water. For patients unable to swallow the tablets whole, e.g. very young children, the tablets can be dissolved in water before administration. The tablets can also be crushed and administered with water. The MB treatment will be provided on a spoon with local food to increase the acceptability for the

children. The PQ tablets will be dissolved in drinking water and given with a plastic cup or spoon, also together or after some food.

Justification: Coarsucam® in the given dosing schedule is the standard of care for the treatment of uncomplicated malaria in Burkina Faso. MB has been used for treatment of young children in Burkina Faso in a number of studies in recent years with dosing regimens ranging from 4 - 24 mg per kg per day over a three days period (twice daily applications with doses ranging from 2 - 12 mg per kg), without any obvious safety problem. Daily MB dosing between 12 and 24 mg per kg was demonstrated to be safe and effective in a dose finding study. The half-life being 20 hours, MB in the dosage of 15 mg per kg per day given as single dose has recently been shown to be safe and effective.

The MB minitablets are a newly developed solid formulation developed at the Heinrich Heine University Düsseldorf in Germany and produced by the company Pharbil Waltrop GmbH in Waltrop, Germany, under GMP conditions. The formulation is based on the recently introduced principle of orodispersible minitablets that dissolve rapidly - within less than 10 seconds - in the oral cavity which prevents aspiration / inhalation of particles (Stoltenberg 2011). The produced mini-tablets are directly compressed, round, biconvex, blue minitablets of 2 mm in diameter and height and contain 2.0 mg Methylene Blue as the active pharmaceutical ingredient. Further ingredients are the following excipients of pharmaceutical grade: Ludiflash™ (containing mannitol, crospovidone and polyvinyl acetate) and magnesium stearate. For Ludiflash™, the manufacturer and supplier has released a paediatric dossier showing the applicability of the ingredients in the paediatric population at all age groups (Hebestreit 2010). Magnesium stearate is generally regarded as safe (GRAS status), as well for paediatric use. Recent data from the University of Düsseldorf show that the use of mini-tablets in children below 6 years is not only safe, but even better accepted than the use of sweet, well-tasting liquids (Klingmann 2015). The new concepts with multiparticulates manufactured on simple, standard pharmaceutical equipment (mixer and rotary tablet machine) fits perfectly with the recently published recommendations of WHO initiative "Making medicines child-size". For the present study, the Methylene Blue mini-tablets should be dispersed in a semi-solid food stuff such as pudding or yoghurt. When suspended in the semi-solid food, the mini-tablets mainly keep the geometry without disintegration, but they remarkably soften and are neither recognised as solid drug carriers in the mouth nor show any risk of aspiration.

The control group will receive once daily one tablet of the fixed dose AS-AQ (Coarsucam®, Sanofi Aventis) using three weight groups (6.0 – 8.9 kg: 25 mg AS + 67.5mg

AQ; 9.0-17.9 kg: 50 mg AS + 135 mg AQ; >17.9 kg: 100 mg AS + 270 mg AQ), given over a three days period. In addition, on day 2 the children of this group will receive a single dose of 0.25 mg/kg PQ (Sanofi, India) using four weight groups (6.0 – 8.9 kg: 2 mg PQ; 9.0-12.9 kg: 3 mg PQ; 13.0-16.9 kg: 4 mg PQ; >17.0 kg: 5 mg PQ).

All children having fever (temperature ≥ 38.5°C) will receive standard doses of paracetamol (= acetaminophen; 10 mg/kg every 6 hours) until symptoms subside.

Study children developing severe malaria during the trial will be admitted to the Nouna hospital and treated with quinine according to national guidelines. Study children developing uncomplicated malaria recrudescence or reinfection or who show asymptomatic parasitological failure during follow-up between day 7 and day 28 will be treated with artemether-lumefantrine according to national guidelines.

Drug storage and drug accountability

The investigator, or his designated deputy (e.g. nurse or pharmacist), will make sure that the Investigational Products are adequately stored in a secured area with restricted access, under recommended storage conditions by the manufacturer and in accordance with applicable regulatory requirements. Prior to dispensing, all study medications should be stored by the investigator between 20 °C and 30 °C. The investigator must record the receipt and usage of all drugs supplied.

A drug accountability utilisation log will be maintained for clinical trial material by the investigator. This will include the date and time of administration, the quantity administered and the patient randomisation number. The log should be signed and dated by the investigator.

Permitted and not permitted concomitant treatments

All indicated drugs are allowed, except other western malaria drugs including antibiotics with malaria efficacy (e.g. cotrimoxazole, doxycycline, acythromycine) and except serotonin reuptake inhibitors (e.g. citalopram, escitalopram, fluoxetin, paroxetine, sertraline) and drugs known to inhibit the liver enzymes cytochrome 2A6 (e.g. methoxsalen, pilocarpine, tranylcypromine) and/or cytochrome 2C8 (e.g. trimethoprim, ketoconazole, ritonavir, saquinavir, lopinavir, gemfibrozil, montelukast) from beginning of treatment until end of follow-up.

Prior and concomitant illnesses

Prior illnesses based on self-reporting of parents/caretakers will be recorded as relevant to the current condition, mainly for the last 7 days. All concomitant illnesses will be recorded based on the examination of the study physicians during the 28 days observation period. Concomitant illnesses between visits will be recorded based on reports from interim hospital visits of study children and/or based on self-reporting of parents/caretakers.

Procedures for monitoring subject compliance

Drug intake will be directly observed and assisted by the study nurses or specific field staff for the whole treatment period. In case of vomiting within 1/2 hour after first drug ingestion, the treatment dose will be repeated. In case of further vomiting, the child will be withdrawn from the study and treated according to local standards.

Visits and follow-up

All parents/caretakers are informed that they should return to the study centres for further examination and treatment in case that the child again develops a significant illness between the first treatment dose and the final visit on day 28. The addresses of all study subjects will be recorded at enrolment of study children, and all children having not returned for one of the scheduled follow-up visits will be visited at home.

Methods of data collection

Haemoglobin (day 0, day 1, day 2, day 3, day 7, day 14, day 28, day X), parasitaemia (day 0, day 1, day 2, day 3, day 7, day 14, day 28, day X), and filter paper samples for determination of G6PD status (day 0), PCR genotyping of malaria parasites (day 0, day 7, day 14, day 28, day X) will systematically be collected (day X is any unscheduled visit between day 3 and day 28). Whenever possible, finger prick blood samples will be taken instead of venous blood samples. Temperature will systematically be measured during all time points with electronic thermometers. On day 7, 14, and 28, self-reported signs and symptoms and any traditional and western treatment taken will systematically be recorded.

Study Plan

	Inclusion/ Treatment			Follow up				Unscheduled visit
	**D0**	**D1**	**D2**	**D3**	**D7**	**D14**	**D28**	**Day X**
**Informed consent**	•							
**Physical Examination**	•	•	•	•	•	•	•	•
**Inclusion/exclusion criteria**	•							
**Blood smears**	•	•	•	•	•	•	•	•
**PCR samples for**								
G6PD status	•							
recrudescence/reinfection	•				•	•	•	•
**Study drugs**	•	•	•					
**Concomitant medication**	•	•	•	•	•	•	•	•
**Adverse events**	•	•	•	•	•	•	•	•
**Haematology**	•	•	•	•	•	•	•	•
**Fever**	•	•	•	•	•	•	•	•

7 Assessment of primary endpoint

- Haemoglobin values will be determined by measurement with HemoCue® at baseline and on day 7.

8 Assessment of secondary endpoints

- Gametocyte prevalence and density will be measured microscopically at baseline and on day 1, 2, 3, 7, 14, and 28 of follow-up
- Parasite clearance time (time between inclusion and clearance of parasites), measured through microscopic determination on day 1, day 2, and day 3.
- Fever clearance time (time between inclusion and clearance of fever), measured with electronic thermometers on day 1, day 2, and day 3.
- ACPR, ETF, LPF and LCF will be determined over the 28 days study period according to the standard WHO protocol (WHO 2003)
- Reports of observed or self-reported adverse event (definition below)
- Reports of patients with at least one serious adverse event:
    1. Acute haemolysis (definition: haemoglobin ≤ 5 g/dl, or received blood transfusion according to clinical judgement of study physician)
    2. Other serious adverse events (definition below)
- Change in haemoglobin after 2, 3, 14, 28 days compared to baseline
- Acceptance of the different treatment regimens by mothers/caretakers

Methods of data collection

Data on the occurrence of adverse events (severity, seriousness and causality), malaria recrudescence/reinfection, concomitant illnesses and concomitant drugs will be collected through continuous active and passive clinical surveillance (including laboratory results) of all study subjects.

The following laboratory methods will be employed:

Haemoglobin concentrations will be measured in the field using a HemoCue® (HemoCue® AB, Angelholm, Sweden), and malaria parasites will be diagnosed with standard techniques by light microscopy (laboratory of CRSN).

Differentiation of recrudescences from new infections will be achieved by comparing PCR-generated *msp1* and *msp2* genotype patterns in matched pairs of isolates obtained on admission and precisely on the day of reappearance of parasitaemia, also based on filter paper blood samples (Snounou et al. 1999) (Laboratory of Tropical Institute Berlin).

The common West African G6PD deficiency allele GdA- will be distinguished from GdA and GdB by PCR methodology, also based on filter paper blood (Laboratory of Tropical Institute Berlin).

An overall statistical analysis plan will be prepared before the database is closed.

Adverse event (AE) definition

The term “adverse event” covers any sign, symptom, syndrome and illness that appears or worsens in a subject during the period of observation in the clinical trial and that may impair the well-being of the subject. The term also covers laboratory findings or results of other diagnostic procedures that are considered to be clinically relevant.

No causal relationship with the study medication is implied by the use of the term “adverse event”. Adverse events fall into the categories of non-serious and serious.

Serious adverse event (SAE) definition (GCP)

A serious adverse event is defined as any untoward medical occurrence at any dose:
(1) results in death, (2) is life-threatening, 3) requires inpatient hospitalisation or prolongation of existing hospitalisation, (4) results in persistent or significant disability/incapacity (5) is a congenital anomaly / birth defect. (“Life-threatening” refers to an event in which the patient

was at risk of death at the time of the event; it does not refer to an event which hypothetically might have caused death if it was more severe).

Classification of severity

Mild: The adverse event does not interfere with the routine activities. The patient may experience slight discomfort.

Moderate: The adverse event interferes with routine activities. The patient may experience significant discomfort.

Severe: The adverse event makes it impossible to perform routine activities. The patient may experience intolerable discomfort or pain.

Classification of causality

Unrelated, possibly related, probably related, definitely related; cannot be assessed, for instance due to lack of information.

Unexpected adverse reaction (UAR) (EU Directive 2001/20/EC of 4 April 2001)

An adverse reaction, the nature or severity of which is not consistent with the applicable product information.

Period of observation of adverse events

This is the period between the informed consent and the final examination on day 28.

Documentation of adverse events and serious adverse events

All adverse and serious adverse events will be recorded on specific forms. The adverse event form contains a detailed description of the adverse event (type of event, beginning and duration, severity, outcome, causality to the study medication, specific therapy), serious adverse event (yes/no), and date and signature of the attending physician. For serious adverse events (SAEs) a description of the SAE and consequences for the trial are documented.

Reporting of serious adverse events

Serious adverse events have to be reported by the attending physician to the principle investigators within 24 hours or not later than the next working day. The SAE is also reported to both of the independent Ethic Committees (Heidelberg and Burkina Faso) as per their requirements.

Early termination of the trial

Reasons that may require the termination of the trial include the following: (1) Incidence of adverse events indicates a potential health hazard caused by the study treatment. (2) It appears that participant's enrolment or study logistics are unsatisfactory. (3) External evidence that makes it necessary to terminate the trial. The steering committee will decide on an early termination.

9 Statistics

Analysis sets

The primary efficacy analysis will be based on the full analysis set (FAS) according to the intention-to-treat (ITT) principle, reflecting the recommendations given in guidelines (e.g. ICH Harmonised Tripartite Guideline, 1999). Furthermore, in 2005 Brittain and Lin demonstrated that the ITT and the per-protocol (PP) set yield almost identical results. Thus, ITT should be preferred as the primary efficacy set here. Additionally, an evaluation of the primary outcome will be performed in the PP set, in accordance to Snapinn in 2000, where the importance of the PP set in non-inferiority trials was emphasised. Before database closure the assignment of each patient to the PP and the ITT population are defined in the statistical analysis plan.

The safety set will consist of all patients who received one of the study treatments at least once, and patients will be allocated to the treatment they actually received. This will be the primary analysis set for the safety analysis.

Confirmatory analysis

The hypotheses for the primary efficacy analysis are

$$H_0: \mu_{PQ} - \mu_{MB} \geq \delta \quad vs. \; H_1: \mu_{PQ} - \mu_{MB} < \delta$$

(δ=0.7, non-inferiority margin), where μ_{MB} and μ_{PQ} are the mean differences in haemoglobin between day 7 and baseline for the AS-AQ-MB and the AS-AQ-PQ group, respectively. Non-inferiority of AS-AQ-MB as compared to AS-AQ-PQ will be tested at one-sided significance level of α=0.025. Missing data for the primary outcome variable will be replaced using multiple imputation (van Buuren, 2012) taking the covariates treatment group and baseline haemoglobin into account by application of the fully conditional specification method (van Buuren, 2007).This will be realised using the option "FCS" of the SAS "MI" procedure which is

implemented in SAS 9.4. Sensitivity analyses will be performed by applying alternative methods dealing with missing data such as, e.g. complete case analysis and replacement by ICA-r (Higgins, 2008). Additionally, a linear regression model will be fitted for a comparison of the mean haemoglobin differences rates between the treatment groups adjusting for baseline haemoglobin.

Analysis of secondary endpoints and blood concentrations

All secondary efficacy variables and the blood concentrations will be analysed descriptively by tabulation of the measures of the empirical distributions. According to the scale level of the variables, means, standard deviations, medians, 1st and 3rd quartiles as well as minimum and maximum or absolute and relative frequencies, respectively, will be reported. Additionally, for variables with longitudinal measurements the time course for individual patients and for the treatment groups will be displayed. Descriptive *p*-values of the corresponding statistical tests comparing the treatment groups and associated 95% confidence intervals will be given.

Safety analysis

Adverse and Serious Adverse Events will be tabulated and absolute and relative frequencies with 95% confidence intervals will be calculated. The severity and the relationship to the treatment will be given and compared between the treatment groups.

Homogeneity of treatment groups

The homogeneity of the treatment groups will be described by comparison of the demographic data and the baseline values by means of descriptive statistics. All analyses will be done using SAS version 9.4 or higher.

10 Trial monitoring and auditing

Clinical Monitoring will be performed by a qualified physician from the National Malaria Research Institute in Ouagadougou, Burkina Faso, an institution which is independent from other trial staff to ensure patient safety and integrity of the clinical data, e.g. primary endpoint in adherence to study protocol. Regular on-site monitoring visits are planned depending on the recruitment rate and quality of the data. The investigator must allow the

monitor to have access to all essential documents and source data and must provide support at all times to the monitor. Existence, informed consent, age and body weight of patients, SAEs and haemoglobin values at baseline and at day 7 will be monitored 100%.

11 Ethical and legal aspects

The protocol will be reviewed by the Ethics Committee of Heidelberg University and the responsible Ethics Committee in Burkina Faso. The trial will be conducted in accordance with local laws and the internationally established principles for GCP (ICH-GCP, 1996; CIOMS, 2002).

Community consent will be sought from the local authorities prior to the selection of study children. The population will be informed of the risks and benefits of the project through sub-district meetings. Written informed consent will be sought from parents/caretakers before the start of the study. All parents/caretakers will be told that they have the right to withdraw their child from the study at any time point without negative consequences. All acute disease episodes of study children will be treated free of charge and according to the local standards of care during the pre-determined follow-up period. Findings of the study will be shared not only with the local and national health authorities, but also with the population.

Data transport to Germany will take place according to established standard rules between the Nouna Health Research Centre and Heidelberg University. Patient data in the CRF will be pseudonymised. Data processing in Germany will be conducted according to German laws.

12 Documentation and use of trial findings

Data management will be performed at the CRSN in Nouna, with specific support from Heidelberg. Double data entry will be performed. Queries will be formulated and resolved in co-operation with the data management unit of the Institute of Medical Biometry and Informatics of Heidelberg University. Data analysis will be done in Heidelberg after the data base has been closed.

13 References

Adjalley S. H., et al. (2011) Quantitative assessment of Plasmodium falciparum sexual development reveals potent transmission-blocking activity by methylene blue. Proc Natl Acad Sci USA. 108(47): E1214-1223.

Aeschlimann C, Küpfer A, Schefer H, Cerny T (1998) Comparative pharmacokinetics of oral and intravenous ifosfamide/mesna/methylen blue therapy. Drug Metabolism and Disposition. 26, 883-890.

Akoachere M, Buchholz K, Fischer E, Burhenne J, Haefeli WE, Schirmer RH, Becker K (2005) In vitro assessment of methylene blue on chloroquine sensitive and resistant Plasmodium falciparum strains reveals synergistic action with artemisinins. Antmicrobiol Agents Chemotherapy. 49: 4592-97.

Anstay NM, Hassanali MY, Mlalasi J, Manyenga D, Mwaikambo ED (1996) Elevated levels of methaemoglobin in Tanzanian children with severe and uncomplicated malaria. Trans R Soc Trop Med Hyg. 90, 147-151.

Bountogo M, Zoungrana A, Coulibaly B, Klose C, Mansmann U, Mockenhaupt F, Burhenne J, Mikus G, Walter-Sack I, Schirmer RH, Sié A, Meissner P, Müller O (2010) Short communication: Efficacy of methylene blue monotherapy in semi-immune adults with uncomplicated falciparum malaria: a controlled trial in Burkina Faso. Tropical Medicine and International Health. 15: 713-17.

Bousema, T and Drakeley C. (2011) Epidemiology and infectivity of Plasmodium falciparum and Plasmodium vivax gametocytes in relation to malaria control and elimination. Clin Microbiol Rev. 24(2): 377-410.

Brittain E, Lin D. (2005) A comparison of intent-to-treat and per-protocol results in antibiotic non-inferiority trials. Stat Med. 24(1):1-10.

Coulibaly B, Pritsch M, Bountogo M, et al. (2015) Efficacy and safety of triple combination therapy with artesunate-amodiaquine-methylene blue for falciparum malaria in children: a randomized controlled trial in Burkina Faso. J Infect Dis. 211(5): 689-697.

Coulibaly B, Zoungrana A, Mockenhaupt F, Schirmer H, Klose C, Mansmann U, Meissner P, Müller O. (2009) Strong gametocytocidal effect of methylene blue-based combination therapy against falciparum malaria: a randomised controlled trial. PLoS One. 4(5):e5318

Dale HH. (1956) Introduction. In Himmelweit F, Marquardt M, Dale H (ed): The collected papers of Paul Ehrlich. Pergamon Press, London.

Davioud-Charvet E, Delarue S, Christophe Biot C, et al. (2001) A prodrug form of a *Plasmodium falciparum* glutathione reductase inhibitor conjugated with an antimalarial 4-anilinoquinoline. J Med Chem. 44, 4268-4276.

DiSanto AR, Wagner JG. (1972) Pharmacokinetics of highly ionized drugs II: methylene blue – absorption, metabolism and excretion in man and dog after oral administration. J Pharm Sci. 61: 1086-1090.

Ehrlich P. (1913) Chemotherapeutics: scientific principles, methods, and results. The Lancet. 445-451

Färber PM, Arscott LD, Williams CH Jr, Becker K, Schirmer RH. (1998) Recombinant Plasmodium falciparum glutathione reductase is inhibited by the antimalarial dye methylene blue. FEBS Lett. 423: 311-314.

Ferreira C (1893) Sur l´emploi du bleu de méthylène dans la malaria infantile. Revue de Thérapeutique Médico-Chirugicale. 1 : 488-525.

Galili Y, Kluger Y, Mianski Z, et al. (1997) Methylene blue – a promising modality in sepsis induced by bowel perforation. Eur Surg Res. 29(5): 390-395

Greenwood BM, Bojang K, Whitty JM, Targett GAT. (2005) Malaria. The Lancet. 365(9469): 1487-98.

Guttmann P, Ehrlich P. (1891) Über die Wirkung des Methylenblau bei Malaria. Berliner Klinische Wochenschrift. 39: 953-956.

Hebestreit P, Oswald F, Widmaier R, Herting M (2010) Ludiflash as excipient for paediatric use. Poster presentation, 2nd EuPFI conference, Berlin.

Higgins JPT, White IR, Wood AM (2008) Imputation methods for missing outcome data in meta-analysis of clinical trials. Clinical Trials 5, 225-239

ICH Harmonised Tripartite Guideline. (1999) Statistical principles for clinical trials. International Conference on Harmonisation E9 Expert Working Group. Stat Med. 18(15):1905-42.

International Artemisinin Study Group. (2002) Artesunate combinations for treating uncomplicated malaria: a prospective individual patient data meta-analysis. In: The Cochrane Library, Issue 2, Oxford.

Kanzok SM, Schirmer RH, Türbachova I, Iozef R, Becker K. (2000) The thioredoxin system of the malaria parasite P. falciparum. Glutathione reduction revisited. J Biol Chem. 275(51): 40180-40186.

Klingmann V, Seitz A, Meissner T, Breitkreutz J, Moeltner A, Bosse HM. (2015) Acceptability of Uncoated Mini-Tablets in Neonates. A Randomized Controlled Trial. J Pediatr. 167(4):893-896.e2.

Mandi G, Witte S, Meissner P, Coulibaly B, Mansmann U , Rengelshausen J et al. (2005) Safety of the combination of chloroquine and methylene blue in healthy adult men with G6PD deficiency from rural Burkina Faso. Tropical Medicine and International Health. 10(1): 32-38.

Mansuri A, Lurie AA. (1993) Concise Review: Methaemoglobinemia. American Journal of Hematology. 42(1): 7-12.

Meissner P, Mandi G, Witte S, Coulibaly B, Mansmann U, Rengelshausen J, Schiek W, Jahn A, Sanon M, Tapsoba T, Walter-Sack I, Mikus G, Burhenne J, Riedel KD, Schirmer H, Kouyaté B, Müller O. (2005) Safety of the methylene blue plus chloroquine combination in the treatment of uncomplicated falciparum malaria in young children of Burkina Faso. Malaria Journal. 4(1): 45.

Meissner P, Mandi G, Coulibaly B, Witte S, Tapsoba T, Mansmann U, Rengelshausen J, Schiek W, Jahn A, Walter-Sack I, Mikus G, Burhenne J, Riedel KD, Schirmer H, Kouyaté B, Müller O. (2006) Methylene blue for malaria in Africa: results from a dose-finding study in combination with chloroquine. Malaria Journal. 5(1): 84-88.

Mueller O, et al. (2003). Clinical efficacy of chloroquine in young children with uncomplicated falciparum malaria - a community-based study in rural Burkina Faso. Tropical Medicine & International Health. 8(3): 202-203.

Müller O, Becher H, van Zweeden AB, Ye Y, Diallo D, Konate A, Gbangou A, Kouyate B, Garenne M. (2001) Effect of zinc supplementation on malaria morbidity among west African children: a randomized double-blind placebo-controlled trial. British Medical Journal. 322: 1567-1572.

Müller O, Sié A, Meissner P, Schirmer RH, Kouyaté B (2009) Artemisinin resistance on the Thai-Cambodian border. The Lancet. 374: 1418-1419.

Müller O, Mockenhaupt FP, Marks B, Meissner P, Coulibaly B, Kuhnert R, Buchner H, Schirmer RH, Walter-Sack I, Sié A, Mansmann U. (2013). Haemolysis risk in methylene blue treatment of G6PD sufficient and G6PD deficient West-African children with uncomplicated falciparum malaria: a synopsis of four RCTs. Pharmacoepidemiology and Drug Safety. 22(4): 376-385.

Noedl H, Youry S, Schaechter K, Smith BL, Duong S, Fukuda MM (2008) Evidence of artemisinin-resistant malaria in Western Cambodia. New Engl J Med 359: 2619-20

Nosten F, Brasseur P. (2002) Combination therapy for malaria – the way forward? Drugs. 62(9): 1315-1329.

Pelgrims J, De Vos F, Van den Brande J, et al. (2000) Methylene blue in the treatment and

prevention of ifosfamide-induced encephalopathy: report of 12 cases and review of the literature. British Journal of Cancer. 82(2): 291-294.

Peter C, Hongwan D, Küpfer A. (2000) Pharmacokinetics and organ distribution of intravenous and oral methylene blue. Eur J Clin Pharmacol. 56(3): 247-250.

Recht J, Ashley E, White N. (2014). Safety of 8-aminoquinoline antimalarial medicines: WHO. ISBN 978 92 4 150697 7.

Rosenthal P J. (2001). Antimalarial Chemotherapy, Mechanisms of Action, Resistance, and New Directions in Drug Discovery. Humana Press.

Sarma GN, Savvides SN, Becker K, Schirmer M, Schirmer RH, Karplus PA (2003) Glutathione reductase of the malarial parasite Plasmodium falciparum: Crystal structure and inhibitor development. J Mol Biol. 328(4): 893-907.

Schirmer RH, Coulibaly, B, Schiek W et al. (2003). Methylene blue in the treatment of malaria – past and presence. Redox Report. 8(5): 272-275.

Schirmer RH, Adler H, Pickhardt M, et al. (2011). "Lest we forget you--methylene blue..." Neurobiol Aging. 32(12): 2325 e7-16.

Snapinn SM. (2000) Noninferiority trials. Current controlled trials in cardiovascular medicine. 1(1):19-21.

Snounou G, Zhu X, Siripoon N, et al. (1999) Biased distribution of msp1 and msp2 allelic variants in Plasmodium falciparum populations in Thailand. Transactions of the Royal Society of Tropical Medicine and Hygiene. 93(4): 369-374.

Snow RW, Craig M, Deichmann U, Marsh K. (1999) Estimating mortality, morbidity and disability due to malaria among Africa`s non-pregnant population. Bulletin of the World Health Organisation. 77(8): 624-640.

Stoltenberg I, Breitkreutz J. (2011) Orally disintegrating mini-tablets (ODMTs) – A novel solid dosage form for paediatric use. European Journal of Pharmaceutics and Biopharmaceutics. 78(3): 462-469.

Van Buuren S. (2007) Multiple imputation of discrete and continuous data by fully conditional specification. Statistical methods in medical research. 16(3):219-42.

Van Buuren S. (2012) Flexible Imputation of Missing Data. Boca Raton: Chapman & Hall/CRC.

Vennerstrom JL, Makler MT, Angerhofer CK, Williams JA. (1995) Antimalarial dyes revisited: xanthenes, azines, and thiazines. Antimicrobial Agents and Chemotherapy. 39(12): 2671-2677.

Wainwright M, Amaral L. (2005) The phenothiazinum chromophore and the evolution of

antimalarial drugs. Tropical Medecine & International Health. 10(6): 501-511.

Walter-Sack I, Rengelshausen J, Oberwittler H, Burhenne J, Müller O, Meissner P, Mikus G. (2009) High absolute bioavailability of methylene blue given as an aqueous oral formulation. Eur J Clin Pharmacol. 65(2): 179-189.

WHO. (2000) Severe malaria. Trans R Soc Trop Med Hyg. 94 suppl 1.

WHO. (2003) Assessment and monitoring of antimalarial drug efficacy for the treatment of uncomplicated falciparum malaria. Ref. number: WHO/HTM/RBM/2003.50.

WHO. (2015). World Malaria Report 2015. ISBN 978 92 4 156515 8.

Zoungrana A, Coulibaly B, Sié A, et al. (2008) Safety and efficacy of methylene blue combined with artesunate or amodiaquine for uncomplicated falcipqarum malaria: a randomized controlled trial from Burkina Faso. PLoS One. 3(2): e1630.

Zulian GB, Tullen E, Maton B. (1995) Methylene blue for ifosfamide-associated encephalopathy. The New England Journal of Medicine. 332(18): 1239-1240.
