# Supplementary material for: Safety and efficacy of artesunate-amodiaquine combined with either methylene blue or primaquine in children with falciparum malaria in Burkina Faso: A randomized controlled trial
Source: PLoS One. 2019 Oct 10;14(10):e0222993. doi: 10.1371/journal.pone.0222993 (PMC6786573; doi:10.1371/journal.pone.0222993)
Supplement: S1 Dataset — (ZIP) [file pone.0222993.s003.zip › data_description.rtf]

MB-PQ-Nouna Trial      

 CONTENTS 	


Dataset name	Description	
AE	Adverse events	
BASE	Baseline	
FAILURE_RESPONSE	Failure/Response	
G6PD	G6PD	
TERMINATION	Premature trial termination	
VISIT	Visit day 0, 1, 2, 3, 7, 14, 28	


Dataset AE  


Variables in Creation Order	
#	Variable	Type	Len	Format	Informat	Label	
1	id	Num	8				
2	ANO	Num	8			ANO	
3	DESCRIP	Char	255	$255.	$255.	AE description	
4	SAE	Num	8	11.	11.	Serious Adverse Event	
5	ONGO	Num	8	11.	11.	Ongoing	
6	SEVERE	Num	8	11.	11.	Severity	
7	OUTCOME	Num	8	11.	11.	Outcome	
8	CAUSAL	Num	8	11.	11.	Causality	
9	ACTION	Num	8	11.	11.	Action taken	
10	THERAPY	Num	8	11.	11.	Therapy	
11	code	Num	8			code	
12	aeonsetday	Num	8			Day of AE onset	
13	aestopday	Num	8			Day of AE stop	


Dataset Base

Variables in Creation Order	
#	Variable	Type	Len	Format	Informat	Label	
1	id	Num	8				
2	sex	Num	8	11.	11.	Sex	
3	weight	Num	8			Weight[kg]	
4	epiday	Num	8			Length of current disease episode [days]	
5	treat	Num	8	4.	4.	Prior treatment of current disease episode	
6	illness	Num	8	4.	4.	Any other prior illnesses within last 7 days	
7	in1	Num	8	4.	4.	6-59 months old child	
8	in2	Num	8	4.	4.	Weight >=6 kg	
9	in3	Num	8	4.	4.	Uncomplicated malaria caused by P.falciparum	
10	in4	Num	8	4.	4.	Asexual parasites >= 2000/µl and <= 100000/µl	
11	in5	Num	8	4.	4.	Axillary temperature >= 37.5 °C or a history of fever during last 24 hours	
12	in6	Num	8	4.	4.	Burkinabe nationality	
13	in7	Num	8	4.	4.	Permanent residence in the study area	
14	in8	Num	8	4.	4.	Written informed consent of parents or care takers	
15	ex1	Num	8	4.	4.	Severe malaria	
16	ex2	Num	8	4.	4.	Mixed malaria infection	
17	ex3	Num	8	4.	4.	Vomiting	
18	ex4	Num	8	4.	4.	Any apparent significant disease, incl. severe malnutrition	
19	ex5	Num	8	4.	4.	A history of a previous, significant adverse reaction or known allergy to one or more of the study drugs	
20	ex6	Num	8	4.	4.	Anaemia	
21	ex7	Num	8	4.	4.	Treated in the same trial before	
22	ex8	Num	8	4.	4.	All modern antimalarial treatment prior to inclusion	
23	ex9	Num	8	4.	4.	Therapy with serotonin reuptake inhibitors	
24	ex10	Num	8	4.	4.	Simultaneous participation in another investigational study	
25	ex11	Num	8	4.	4.	Treatment with other investigational drugs	
26	ex12	Num	8	4.	4.	Patient with known HIV/AIDS disease	
27	ex13	Num	8	4.	4.	Therapy with drugs known to inhibit liver enzymes cytochrome 2A6 and/or 2C8	
28	treatment	Num	8			Treatment group	
29	age_group	Num	8			Age (<=36months/>36months)	


Dataset Failure_Response

Variables in Creation Order	
#	Variable	Type	Len	Label	
1	id	Num	8		
2	etf	Num	8	Early treatment failure	
3	lcf	Num	8	Late clinical failure	
4	lpf	Num	8	Late parasitological failure	
5	acpr	Num	8	Adequate clinical and parasitological response	
6	acpr_adj	Num	8	Adequate clinical and parasitological response - adjusted for reinfection	
7	etf_adj	Num	8	Early treatment failure - adjusted for reinfection	
8	lcf_adj	Num	8	Late clinical failure - adjusted for reinfection	
9	lpf_adj	Num	8	Late parasitological failur - adjusted for reinfection	


Dataset G6PD


Variables in Creation Order	
#	Variable	Type	Len	Format	Informat	Label	
1	id	Num	8				
2	G6PD_202_Light_SNiP	Char	3	$3.	$3.	G6PD-202 Light SNiP	
3	G6PD_376_Light_SNiP	Char	3	$3.	$3.	G6PD-376 Light SNiP	
4	G6PD_Genotype	Char	4	$4.	$4.	G6PD Genotype	
5	G6PD_phenotype	Char	12	$12.	$12.	G6PD phenotype	
6	Sex_interpretation	Char	11	$11.	$11.	Sex interpretation	


Dataset termination


Variables in Creation Order	
#	Variable	Type	Len	Format	Informat	Label	
1	id	Num	8				
2	REASON	Num	8	11.	11.	Reason for premature termination	
3	VIOL_TERM	Num	8	4.	4.	Protocol violation	
4	termday	Num	8			Day of premature termination	


Dataset visit


Variables in Creation Order	
#	Variable	Type	Len	Format	Informat	Label	
1	id	Num	8				
2	day	Num	8			Day	
3	temp	Num	8			Temperature [°C]	
4	hb	Num	8			Hemoglobin [g/dl]	
5	falci	Num	8	4.	4.	P.falciparum merozoites	
6	falcidens	Num	8			P.falciparum merozoites paras. density	
7	gameto	Num	8	4.	4.	P.falicparum gametocytes	
8	gametodens	Num	8			P.falciparum gamotocytes paras. density	
9	ovale	Num	8	4.	4.	P.ovale	
10	ovaledens	Num	8			P.ovale paras. density	
11	malariae	Num	8	4.	4.	P.malariae	
12	maldens	Num	8			P.malariae paras. density	
13	fg6pd	Num	8	4.	4.	Filterpaper for G6PD deficiency	
14	fgeno	Num	8	4.	4.	Filterpaper for parasite genotyping	
15	_treat	Num	8			Treatment group (given)	
16	act1	Num	8			Dose AS-AQ	
17	mb1	Num	8			Dose MB	
18	pq1	Num	8			Dose PQ	
19	vomit1	Num	8			Vomiting within 1/2 hours after treatment	
20	act2	Num	8			Dose AS-AQ (rep.application)	
21	mb2	Num	8			Dose MB (rep.application)	
22	pq2	Num	8			Dose PQ (rep.application)	
23	vomit2	Num	8			Vomiting after repeated application	
24	ae	Num	8	4.	4.	Adverse event	
25	cmed	Num	8	4.	4.	Concomitant medication	
26	urine	Num	8	4.	4.	Urine sample blue	
27	accept	Num	8	11.	11.	How convenient was the application of study medication	
28	VIOL	Num	8	4.	4.	Any protocol deviation	
